# Clinicopathological and functional evaluation of replication protein A in epithelial ovarian cancers: A target validation study

**DOI:** 10.1016/j.tranon.2026.102709

**Published:** 2026-02-17

**Authors:** Mashael Algethami, Amera Sheha, Nehal Singhania, Shatha Alqahtani, Ahmed Shoqafi, Çağla Tosun, Jake Spicer, Michael S Toss, Adel Alblihy, Ayat Lashen, Jennie N Jeyapalan, Nigel P Mongan, Emad A Rakha, Srinivasan Madhusudan

**Affiliations:** a Naaz-Coker Ovarian Cancer Research Centre, Biodiscovery Institute, School of Medicine, University of Nottingham, University Park, Nottingham NG7 3RD, UK; b Sulaiman AlRajhi University, College of Applied Sciences, Department of Medical Laboratory Sciences, Al-Bukayriyah 51941, Qassim, Saudi Arabia; c Histopathology Department, South Egypt Cancer Institute, Assiut University, Assiut, Egypt; d Department of Pathology, Nottingham University Hospital, City Campus, Hucknall Road, Nottingham NG51PB, UK; e Medical Center, King Fahad Security College (KFSC), Riyadh 11461, Saudi Arabia; f Faculty of medicine and Health Sciences, Centre for Cancer Sciences, University of Nottingham, Sutton Bonington Campus, Sutton Bonington, Leicestershire LE12 5RD, UK; g Department of Pharmacology, Weill Cornell Medicine, NY, NY, 10065, USA; h Department of Oncology, Nottingham University Hospitals, Nottingham NG51PB, UK

**Keywords:** RPA1, RPA2;RPA3, Platinum resistance, PARP inhibitor, Olaparib, Talazoparib, Synthetic lethality, PARP inhibitor resistance, HAMNO

## Abstract

Replication Protein A (RPA), a single-stranded DNA (ssDNA)-binding protein is critically involved in DNA replication, checkpoint regulation and DNA repair. We evaluated RPA1, 2, and 3 sub-units protein expression in 331 ovarian tumours, transcripts in 1287 tumours and bioinformatics in the ovarian TCGA cohort (n = 379). Platinum resistant ovarian cancer cells were depleted for RPA 1 or 2 and tested for cisplatin, olaparib and talazoparib sensitivity. HAMNO (RPA1 protein-protein interaction inhibitor) was tested in sensitive and resistant cells. High nuclear RPA1 and RPA2 protein was significantly associated with high grade serous ovarian cancers (HGSOC), advanced stage, platinum resistance and worse progression free survival (PFS) (all ps <0.05). High *RPA1 and RPA2* transcripts also linked with poor PFS. Preclinically, RPA1 or RPA 2 depletion increased sensitivity to cisplatin, olaparib and talazoparib treatment. HAMNO monotherapy was cytotoxic to sensitive and resistant cells. We conclude that RPA directed precision oncology strategy could be a viable strategy in HGSOC.

## Highlights

• Replication Protein A (RPA), a single-stranded DNA (ssDNA)-binding protein is critically involved in DNA replication, checkpoint regulation and DNA repair.
• Comprehensive protein and transcriptomic evaluation show that RPA overexpression is linked with aggressive ovarian cancer and platinum resistance.
• RPA depletion promoted platinum and PARP inhibitor sensitivity.

## Introduction

High-grade serous ovarian carcinoma (HGSOC) is the commonest cause of gynaecological cancer-related death [1]. Recent advances in surgery, systemic platinum-based chemotherapy, and targeted therapies (PARP inhibitors, anti-angiogenic agents) have improved clinical outcomes [[2], [3], [4], [5]]. However, intrinsic or acquired resistance to current systemic therapies pose considerable clinical challenges [[2], [3], [4], [5]]. Therefore, the development of novel biomarkers and therapeutic targets remain an area of unmet need in HGSOC.

Homologous recombination (HR) pathway is a critical for the repair of DNA double strand breaks (DSBs). Homologous recombination repair deficiency (HRD) leading to genomic instability is a feature of about 50 % HGSOC [6]. Loss of key HR genes such as BRCA1 or BRCA2 either at germline or due to somatic mutations or promoter hypermethylation is observed in up to 30 % of all HGSOCs [7]. In addition to HR function, BRCA1 and BRCA2 also have roles during stalled replication fork repair (SFR) and the suppression of replication stress [8,9]. Importantly, chronic replication stress is commonly observed in HGSOC. Such replication stress may arise due to persistent DSBs due to HRD, transcription-replication conflicts, or amplified oncogenes (including *CCNE1*, and *c-MYC amplifications)* [10]. Long stretches of single strand DNA (ssDNA) are inevitable during replication stress and are common DNA repair intermediates. Replication protein A (RPA) is a ssDNA binding protein that coats and protects exposed ssDNA from endogenous nuclease digestion. RPA is a multi-domain heterotrimeric protein consisting of RPA1 (70 kDa), RPA2 (32 kDa), and RPA3 (14 kDa) sub-units. The protein-protein interactions (PPI) required for the regulation of DNA replication, repair and recombination is performed through RPA1 (N-terminal domain) and RPA2 (C-terminus) sub untis [[11], [12], [13], [14], [15], [16], [17], [18]].

We hypothesised that RPA may influence ovarian cancer pathogenesis and could be a potential anti-cancer drug target. To address this hypothesis, clinicopathological significance of RPA expression was investigated at the protein and transcriptomic level in clinical cohorts of ovarian cancers. Pre-clinically we depleted RPA1/2 in a panel of ovarian cancer cells and tested for platinum and PARP inhibitor sensitivity. We then evaluated HAMNO, a small molecule RPA1 protein-protein interaction inhibitor in platinum and PARPi resistant ovarian cancer cells lines.

## Results

### Clinical studies

#### High RPA protein expression is associated with aggressive cancers

Immunohistochemical staining of RPA1, RPA2 and RPA3 was performed in a cohort of 331 patients with ovarian cancers. Patient demographics is summarized in N.

**Supplementary Table 1**.

**Fig. 1:**
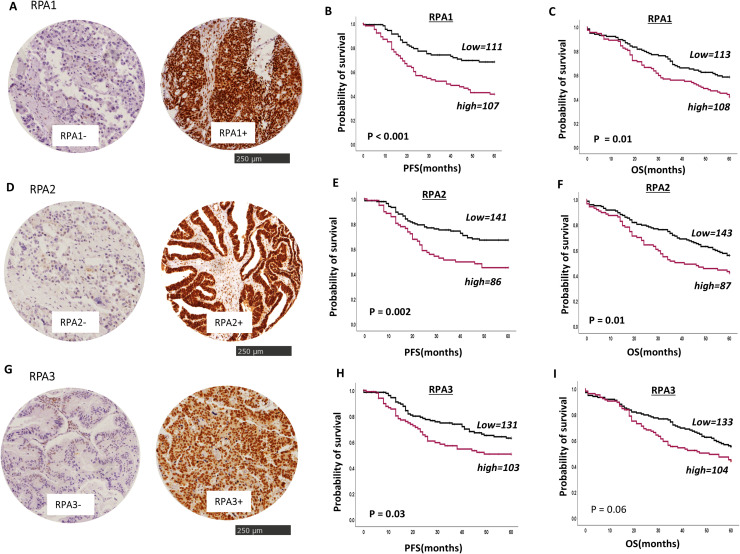
RPA protein expression and clinical ovarian cancers. A. Immunohistochemical (IHC) staining of negative (-) RPA1 nuclear protein expression or positive (+) RPA1 protein expression. B. Kaplan Meier curves of PFS and RPA1 protein expression. C. Kaplan Meier curves of OS and RPA1 protein expression. D. IHC staining of negative (-) RPA1 nuclear expression or positive (+) RPA2 protein expression. E. Kaplan Meier curves of PFS and RPA2 protein expression. F. Kaplan Meier curves of OS and RPA2 protein expression. G. IHC staining of negative (-) RPA1 nuclear expression or positive (+) RPA2 protein expression. H. Kaplan Meier curves of PFS and RPA2 protein expression. I. Kaplan Meier curves of OS and RPA2 protein expression.

**RPA1:** IHC analysis revealed only nuclear staining for RPA1 (Fig. 1A). No cytoplasmic staining for RPA1 was observed. High nuclear RPA1 expression was observed in 49 % (108/219) of tumours. High RPA1 was significantly associated with HGSOC (p = 0.000010), high grade (p = 0.002), high stage (p = 0.004) and platinum resistance (p = 0.016) (Table 1). High RPA1 protein expression was significantly associated with worse progression free survival (PFS) (p<0.0001) (Fig. 1B) and poor overall survival (OS) (p = 0.01) (Fig. 1C).

**Table 1:** RPA expression and clinicopathological associations in ovarian cancers.

	RPA1		X^2^ *P*-value
	Low	High	
**Tumour grade** Low Intermediate High	22(21 %) 28(27 %) 55(52 %)	9(8.2 %) 19(17.3 %) 82(74.5 %)	12.387 **0.002**
**Histopathology type** Serous Mucinous Endometriod Clear cell Mixed Other histological subtypes	53(43 %) 25(20 %) 22(18 %) 12(10 %) 3(2 %) 9(7 %)	89(73 %) 6(5 %) 10(8 %) 5(4 %) 7(6 %) 5(4 %)	30.883 **<0.0001**
**Stage** 1 2 3 4	57(47.5 %) 16(13.3 %) 43(35.8 %) 4(3.3 %)	30(25.4 %) 21(17.8 %) 64(54.2 %) 3(2.5 %)	13.303 **0.004**
**Platinum response** Sensitive Resistant	102(95 %) 5(5 %)	89(86 %) 15(14 %)	5.843 **0.014**

	**RPA2**		X^2^ *P*-value
	Low	High	
**Tumour grade** Low Intermediate High	24(18 %) 34(25 %) 76(57 %)	10(11 %) 13(15 %) 65(74 %)	6.765 **0.034**
**Histopathology type** Serous Mucinous Endometriod Clear cell Mixed Other histological subtypes	76(48.4 %) 30(19.1 %) 24(15.3 %) 14(8.9 %) 6(3.8 %) 7(4.5 %)	74(72.5 %) 4(3.9 %) 9(8.8 %) 4(3.9 %) 8(7.8 %) 3(2.9 %)	23.551 **<0.0001**
**Stage** 1 2 3 4	71(47 %) 21(14 %) 54(36 %) 4(3 %)	27(28 %) 18(18 %) 48(49 %) 5(5 %)	9.986 **0.019**
**Platinum response** Sensitive Resistant	129(94 %) 8(6 %)	72(86 %) 12(14 %)	4.513 **0.031**

	**RPA3**		X^2^ *P*-value
	Low	High	
**Tumour grade** Low Intermediate High	21(17 %) 32(26 %) 68(56 %)	14(13 %) 20(18 %) 75(69 %)	3.896 0.143
**Histopathology type** Serous Mucinous Endometriod Clear cell Mixed Other histological subtypes	73(51 %) 24(17 %) 20(14 %) 12(8 %) 6(4 %) 8(6 %)	78(64 %) 9(7 %) 18(15 %) 8(7 %) 5(4 %) 4(3 %)	7.697 0.174
**Stage** 1 2 3 4	64(47 %) 15(11 %) 55(40 %) 3(2 %)	38(32 %) 24(20 %) 50(42 %) 6(5 %)	8.574 **0.036**
**Platinum response** Sensitive Resistant	120(95 %) 6(5 %)	88(87 %) 13(13 %)	4.807 **0.026**

**RPA2:** We observed nuclear staining only for RPA2 (Fig. 1D). 37 % (87/229) of tumours had high RPA2 expression. High RPA2 expression was highly significantly associated with HGSOC (p < 0.0001), high tumour grade (p = 0.034), advanced stage (p = 0.019), and platinum resistance (p = 0.016) in using a publicly available online gene expression dataset for 1287 patients with ovarian cancer (Table 1). High RPA1 protein expression was significantly linked with poor PFS (p = 0.002) (Fig. 1E) and poor OS (p = 0.01) (Fig. 1F).

**RPA3:** High nuclear RPA3 was seen in 50 % (103/206) tumours (Fig. 1G). High nuclear RPA3 was significantly associated with advanced stage (p = 0.036) and platinum resistance (p = 0.026) (Table 1). High RPA3 expression was associated with poor PFS (p = 0.03) (Fig. 1H) but not OS (p = 0.06) (Fig. 1I).

**PFS and OS based on stage and platinum sensitivity/resistance:** The data is shown in **Supplementary Figures 1, 2, 3 and 4**. In platinum sensitive tumours, low RPA1 (p = 0.008, **Supplementary Figures 3A**) and RPA2 (p = 0.009, **Supplementary Figures 3B**) were significantly associated with better PFS. In platinum resistant disease, high RPA1 (p = 0.01, **Supplementary Figures 4A**) and high RPA3 (p = 0.005 **Supplementary Figures 4C**) were significantly associated with poor PFS.

**Correlation between RPA1, 2 and 3 protein expression:** We observed significant positive correlation between RPA1, RPA2 and RPA3 protein expression as shown in **Supplementary Figure. 5**.

**Multivariate analysis:** As shown in Table 2, RPA1 remained independently associated with PFS (p = 0.01) along with pathology stage (p < 0.001) and platinum sensitivity (p = 0.009). For OS, pathology stage (p < 0.001) and platinum sensitivity (p < 0.001) only remained independently significant.

**Table 2:** Multivariate analysis.

Parameters	Progression free survival			Overall survival		
	HR	95 % CI	p-value	HR	95 % CI	p-value
RPA1	2.64	1.20 – 5.8	**0.01**	1.28	0.61 – 2.7	0.50
RPA2	1	0.49 – 2	0.99	0.64	0.31 – 1.3	0.23
RPA3	0.87	0.41 – 1.8	0.73	1.49	0.68 – 3.2	0.31
Pathology stage	2.18	1.42 – 3.3	**<0.001**	1.96	1.34 – 2.8	**<0.001**
Pathology grade	1.11	0.69 – 1.7	0.66	0.89	0.59 – 1.3	0.58
Residual tumor following surgery	0.92	0.70 – 1.2	0.61	1.07	0.82 – 1.4	0.58
Platinum sensitivity	4.67	1.47 – 14.8	**0.009**	5.22	2.17 – 12.5	**<0.001**
Age	0.66	0.36 – 1.1	0.16	0.95	0.54 – 1.6	0.88

#### High *RPA* transcripts and survival outcomes

**Fig. 2:**
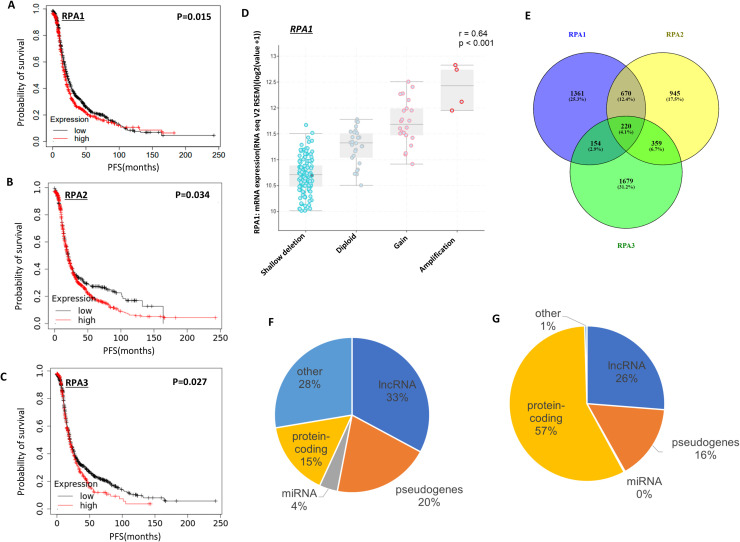
RPA mRNA expression and clinical ovarian cancers. A. Kaplan Meier curves of PFS and RPA1 mRNA expression. B. Kaplan Meier curves of PFS and RPA2 mRNA expression. C. Kaplan Meier curves of PFS and RPA3 mRNA expression. D. Bioinformatic analysis of genetic alterations and differential gene expression profiling for RPA complex components in TCGA-OV cohort. GISTIC analysis of RPA1 genetic alterations compared to mRNA expression levels from RNA-seq data. Pearson correlation coefficient and p-value shown. E. Venn diagram showing comparison of the differential genes identified for tumours with low versus high RPA individual components. F. Graphical representation of the gene-types assigned to the genes expressed higher in Q1 low RPA complex (n = 1787). G. genes expressed higher in Q4 high RPA complex (n = 423). Gene types were lncRNA, miRNA, pseudogenes (including transcribed and processed pseudogenes) and other RNA (tRNA, snoRNA, scaRNA and misc RNA).

As shown in Fig. 2A, high *RPA1* transcripts is associated with poor PFS (p = 0.015) but not OS (p = 0.35) (Supplementary Figure. 6A). High *RPA2* transcripts was linked with poor outcome in terms of shorter PFS (p = 0.034) (Fig. 2B) and OS (p = 0.0007) (Supplementary Figure. 6B). High *RP3* transcripts was also associated with shorter PFS (p = 0.027) (Fig. 2C) and shorter OS (p = 0.0017) (Supplementary Figure. 6C). We did not observe any significant correlation between *RP1, RP2* and *RPA3* transcripts expression as shown in Supplementary Figure. 6D-**F**.

Taken together, protein and transcriptomic data provide evidence that RPA1, 2 and 3 are associated with aggressive features and have prognostic significance in ovarian cancer. We proceeded to conduct detailed bioinformatics study in the TCGA cohort.

#### Bioinformatics

As *RPA1, 2 or 3* downregulation may have a global genome wide impact, we conducted detailed bioinformatics studies. We utilised cBioportal to examine the TCGA-OV cohort (TCGA firehose legacy cohort, n = 316 specimens). Mutations in *RPA1, RPA2* and *RPA3* were rare, with only one specimen harbouring a mutation in *RPA1* (frameshift S609Rfs*46). The copy number variations compared to diploid specimens in *RPA1, RPA2* and *RPA3* were amplifications (>2 copies), gains (gain of 2 copies), with the majority being shallow deletions (loss of a copy). Copy number alterations showed significant positive correlation with mRNA levels for *RPA1* (P = <0.001, Fig. 2D), *RPA2* (P = <0.001, **Supplementary Figure. 7A**) and *RPA3* (P = <0.001, **Supplementary Figure. 7B**).

We then examined the differential gene expression between tumours with low RPA complex mRNA expression and tumours with high RPA complex mRNA expression (Q1 v Q4; **Supplementary file 1**). Genes (n = 2023) that were at a higher level in low RPA complex (Q1) were associated with the KEGG pathways that have histone gene clusters (hsa05322 systemic lupus, hsa05034 Alcoholism and hsa05203 Viral carcinogenesis, all pathways FDR <0.05). Genes expressed higher in high RPA complex tumours (high in Q4 genes n = 435), were associated with neuroactive ligand-receptor interaction pathway (hsa04080, FDR <0.05). To see if changes to the individual RPA components gave similar findings, comparison of the differential genes for *RPA1, RPA2* and *RPA3* was performed (Fig. 2E; Supplementary Files 2–4). The findings showed 220 genes in common only, with the majority of the differential genes expressed at a higher level in low RPA tumours (*RPA1* high Q1 n = 1748, high Q4 n = 657; *RPA2* high Q1 n = 1871, high Q4 n = 324; *RPA3* high Q1 n = 1692, high in Q4 n = 720). Interestingly, most of the genes identified within the tumours with higher levels in Q1 (low RPA) were non-coding genes, with only 15 % protein-coding, whereas 57 % of the genes were protein coding for genes expressed higher with high RPA complex (Fig. 2F, 2G). This higher level of non-coding gene expression was also seen for the RPA individual components (**Supplementary Figure. 7C-H**). As we have shown higher levels of RPA to give worse outcomes, we examined the differential genes with higher expression in RPA high tumours. Sineoculis homeobox homolog 1 (SIX1), a member of the SIX family of transcription factors known to play important roles in cell proliferation, differentiation, apoptosis, adhesion, and migration was high in *RPA* high tumours. SIX1 has been shown to play a role in tumours, including in ovarian cancer, and therefore associated with poorer outcomes in ovarian cancer [[19], [20], [21]]. Furthermore, members of the Cut homeobox family of transcription factors (ONECUT1, ONECUT2, and ONECUT3) were also expressed higher in *RPA* high tumours. Previously, *ONECUT2, also known as HNF5B,* was shown to be up regulated in some ovarian cancer cell lines and in malignant ovarian cancer tissue, with silencing this gene leading to decreased cell cancer growth in vitro and in vivo [22]. Interestingly, ONECUT2 has been shown to regulate *VEGF* expression [22], and it has previously been shown to be associated with platinum resistance [23], suggesting a complex role between RPA, ONECUT2, and platinum resistance in ovarian cancer. Taken together, these findings suggest that high RPA complex levels correlated with genes involved in platinum resistance, showing potential involvement in the resistance process.

### Pre-clinical studies

Clinical data presented above provide evidence that RPA1 or 2 protein overexpression is associated with poor progression free survival, a surrogate marker of platinum resistance in patients. To validate if RPA expression is associated with platinum resistance, we proceeded to pre-clinical studies.

### RPA1 depletion reverses platinum resistance

**Fig. 3:**
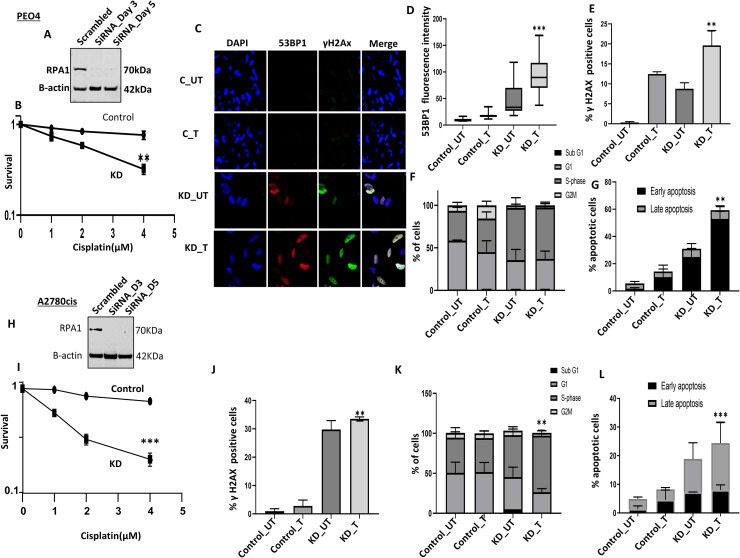
RPA1 depletion and cisplatin sensitivity. A. Representative western blot of RPA1 knock down (KD) using siRNA in PEO4 cells. B. Clonogenic assay of cisplatin sensitivity in control PEO4 and RPA1_KD_PEO4 cells. PEO4 plating efficiency at the highest Cisplatin dose was 18 %. C. Immunofluorescent analysis of 53BP1 and γH2AX in control PEO4 and PEO4_RPA1_KD cells untreated (UT) and treated with cisplatin (5 μM) for 24 hours. D. Quantification of 53BP1 nuclear fluorescence in UT and cisplatin (5 μM) treated control PEO4 and PEO4 _RPA1_KD cells. E. Quantification of γH2AX nuclear fluorescence in UT and cisplatin (5 μM) treated control PEO4 and PEO4 _RPA1_KD cells. F. Cell cycle analysis of UT and cisplatin (5 μM) treated control PEO4 and PEO4_RPA1_KD cells G. Annexin V analysis of UT and cisplatin (5 μM) treated PEO4 and PEO4_RPA1_KD cells. H. Representative western blot of RPA1 knock down (KD) using siRNA in A2780cis cells. I. Clonogenic assay of cisplatin sensitivity in control A2780cis and RPA1_KD_ A2780cis cells. A2780cis plating efficiency at the highest Cisplatin dose was 32 %. J. Quantification of γH2AX nuclear fluorescence in UT and cisplatin (5 μM) treated control PEO4 and PEO4 _RPA1_KD cells. K. Cell cycle analysis of UT and cisplatin (5 μM) treated control A2780cis and A2780cis_RPA1_KD treated cells. L. Annexin V analysis of UT and cisplatin (5 μM) treated control A2780cis and A2780cis_RPA1_KD treated cells. UT = untreated cells; T = cisplatin treated cells. The experiment was performed for samples from three independent experiments (n=3) and the error bar represent the standard deviation (SD). ** p<0.01.

A panel of platinum resistant (A2780cis and PEO4) and platinum sensitive (A2780, PEO1) ovarian cancer cell lines were investigated for basal levels of RPA1 protein expression. As shown in **Supplementary Figures 8A & 8B**, in whole cell lysates, RPA1 was higher in platinum resistant A2780cis and PEO4 cells compared to platinum sensitive A2780 and PEO1 cells. We then depleted RPA1 using siRNAs in PEO4 (Fig. 3A) and A2780cis (Fig. 3H) to investigate platinum sensitivity.

As shown in Fig. 3B, RPA1_KD_PEO4 cells were significantly more sensitive to cisplatin treatment compared to scrambled control cells. To investigate for accumulation of DNA double strand breaks (DSBs), we evaluated 53BP1 foci using immunofluorescent (IF) staining. We observed increased 53BP1 foci in untreated PEO4_RPA1_KD cells compared to untreated control cells implying basal DSB accumulation and increased level of increased genomic instability in PEO4_RPA1_KD cells compared to controls (Fig. 3C, 3D). Following cisplatin treatment, significant accumulation of 53BP1 foci was evident in PEO4_RPA1_KD cells compared to cisplatin treated controls (Fig. 3C, 3D). We also observed similar increase in γH2AX foci in cells (Fig. 3C, Supplementary Figure. 8C). By FACS, similarly, we observed increased γH2AX fluorescence in PEO4_RPA1_KD cells compared to cisplatin treated controls (Fig. 2E). Accumulation of DSBs was associated with increased S-phase accumulation in untreated and cisplatin treated PEO4_RPA1_KD cells compared to untreated, and cisplatin treated controls respectively (Fig. 3F). If DSBs are unrepaired, cell eventually undergo apoptosis. As expected, upon cisplatin treatment, significant accumulation of apoptotic cells was observed in untreated, and cisplatin treated PEO4_RPA1_KD cells compared to untreated, and cisplatin treated controls respectively (Fig. 3G). We then validated these observations in A2780cis cell line. A2780cis_RPA1_KD cells (Fig. 3H) were also significantly more sensitive to cisplatin treatment compared to scrambled control cells (Fig. 3I). We observed increased γH2AX+ cells in untreated A2780cis _RPA1_KD cells compared to untreated control cells as well as in cisplatin treated A2780cis _RPA1_KD cells compared to cisplatin treated controls (Fig. 3J). Accumulation of DSBs was associated with increased S-phase accumulation in untreated and cisplatin treated A2780cis _RPA1_KD cells compared to untreated, and cisplatin treated controls respectively (Fig. 3K). Upon cisplatin treatment, significant accumulation of apoptotic cells was also observed in untreated, and cisplatin treated A2780cis _RPA1_KD cells compared to untreated, and cisplatin treated controls respectively (Fig. 3L). We further validated in another siRNA construct and observed similar cisplatin cytotoxicity potentiation (**Supplementary Figure. 8D, 8E**).

Taken together, the data provides evidence that RPA1 depletion reverses platinum resistance in PEO4 and A2780cis cells.

### RPA1 deficiency increases PARP inhibitor sensitivity

**Fig. 4:**
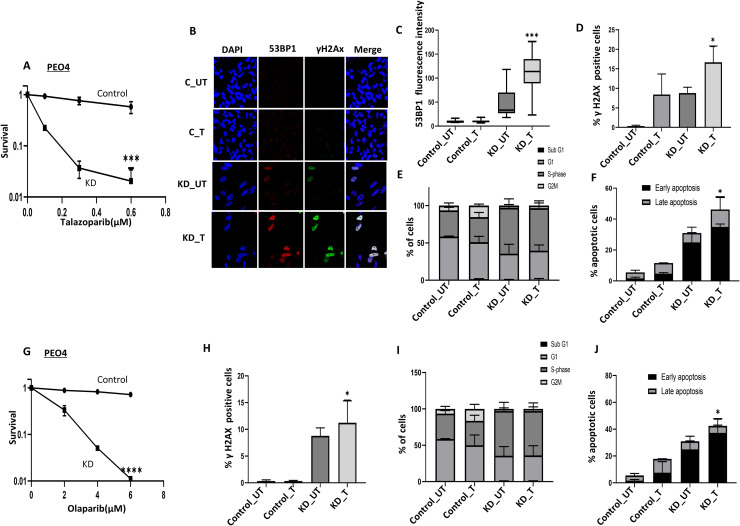
RPA1 depletion and PARP inhibitor sensitivity in PEO4 cells. A. Clonogenic assay of talazoparib sensitivity in control PEO4 and RPA1_KD_PEO4 cells. PEO4 plating efficiency at the highest dose of talazoparib was 10 %. B. Immunofluorescent analysis of 53BP1 and γH2AX in control PEO4 and PEO4_RPA1_KD cells untreated (UT) and treated with talazoparib (800 nM) for 24 hours. C. Quantification of 53BP1 nuclear fluorescence in UT and talazoparib (800 nM) treated control PEO4 and PEO4 _RPA1_KD cells. D. Quantification of γH2AX nuclear fluorescence in UT and talazoparib (800 nM) treated control PEO4 and PEO4 _RPA1_KD cells. E. Cell cycle analysis in UT and talazoparib (800 nM) treated control PEO4 and PEO4_RPA1_KD cells F. Annexin V analysis of UT and talazoparib (800 nM) treated PEO4 and PEO4_RPA1_KD cells. G. Clonogenic assay of olaparib sensitivity in control PEO4 and RPA1_KD_ PEO4 cells. PEO4 plating efficiency at the highest dose of olaparib was 13 %. H. Quantification of γH2AX nuclear fluorescence in UT and Olaparib (6 μM) treated control PEO4 and PEO4 _RPA1_KD cells. I. Cell cycle analysis in UT and Olaparib (6 μM) treated control PEO4 and PEO4_RPA1_KD treated cells. J. Annexin V analysis of UT and Olaparib (6 μM) treated control PEO4 and PEO4_RPA1_KD treated cells. UT = untreated cells; T = talazoparib or olaparib treated cells. The experiment was performed for samples from three independent experiments (n=3) and the error bar represent the standard deviation (SD). ** p<0.01.

As shown in Fig. 4A, control PEO4 is resistant to talazoparib. However, RPA1 depletion led to profound re-sensitivity to talazoparib in RPA1_KD_PEO4 cells (Fig. 4A). Enhanced talazoparib sensitivity was associated with DSB accumulation as evidenced by immunofluorescent (IF) staining showing increased 53BP1 (Fig. 4B, 4C) and increased γH2AX (Fig. 4B, Supplementary Figure. 8F) as well as γH2AX positive cells (Fig. 4D) by FACS. Upon talazoparib treatment, RPA1_KD_PEO4 cells arrested in S-phase compared to talazoparib treated control cells (Fig. 4E). Significant accumulation of apoptotic cells was also evident in talazoparib treated PEO4_RPA1_KD cells compared to talazoparib treated controls respectively (Fig. 4F). We then tested Olaparib sensitivity. As expected, RPA1_KD_PEO4 cells were significantly more sensitive to olaparib treatment than control cells (Fig. 4G). Increased sensitivity was associated with DSB accumulation (Fig. 4H), S-phase arrest (Fig. 4I), and increased apoptosis (Fig. 4J) in PEO4_RPA1_KD cells compared to the control cells.

We further validated in platinum resistant A2780cis cells. As shown in **Supplementary Figure. 9A**, control A2780cis cells are resistant to talazoparib treatment. Whereas RPA1_KD_A2780cis cells are sensitive to talazoparib. Increased sensitivity is associated with increased DSBs (**Supplementary Figure. 9B)**, S-phase arrest (**Supplementary Figure. 9C**) and apoptotic cell accumulation (**Supplementary Figure. 9D)**. Similarly, RPA1_KD_A2780cis cells were also sensitive to olaparib compared to control (**Supplementary Figure. 9E)** and associated with increased DSBs (**Supplementary Figure. 9F)**, S-phase arrest (**Supplementary Figure. 9G**) and apoptotic cell accumulation (**Supplementary Figure. 9H)**.

Taken together, the data suggest that RPA1 could be predictive biomarker of PARP inhibitor resistance. We then depleted RPA2 and investigated cisplatin as well as PARP inhibitor sensitivity in PEO4 and A2780cis cells.

**Fig. 5:**
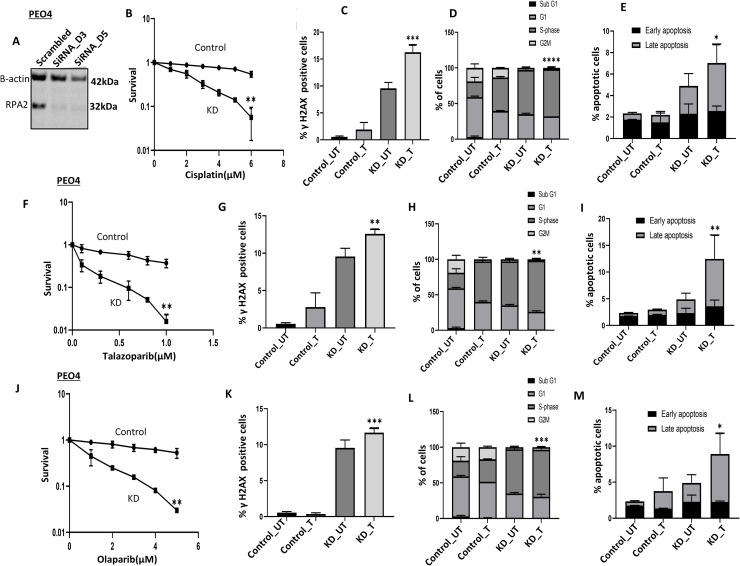
RPA2 depletion and cisplatin or PARP inhibitor sensitivity. A. Representative western blot of RPA2 knock down (KD) using siRNA in PEO4 cells. B. Clonogenic assay of cisplatin sensitivity in control PEO4 and RPA2_KD_PEO4 cells. PEO4 plating efficiency at the highest Cisplatin dose was 18 % C. Quantification of γH2AX nuclear fluorescence in UT and cisplatin (5 μM) treated control PEO4 and PEO4 _RPA2_KD cells. D. Cell cycle analysis of UT and cisplatin (5 μM) treated control PEO4 and PEO4_RPA2_KD cells E. Annexin V analysis of UT and cisplatin (5 μM) treated PEO4 and PEO4_RPA2_KD cells. F. Clonogenic assay of talazoparib sensitivity in control PEO4 and RPA2_KD_PEO4 cells. PEO4 plating efficiency at the highest dose of talazoparib was 10 %. G. Quantification of γH2AX nuclear fluorescence in UT and talazoparib (800 nM) treated control PEO4 and PEO4 _RPA2_KD cells. H. Cell cycle analysis of UT and talazoparib (800 nM) treated control PEO4 and PEO4_RPA2_KD cells I. Annexin V analysis of UT and talazoparib (800 nM) treated PEO4 and PEO4_RPA2_KD cells.F. Clonogenic assay of Olaparib (6 μM) sensitivity in control PEO4 and RPA2_KD_PEO4 cells PEO4 plating efficiency at the highest dose of olaparib was 13 %. G. Quantification of γH2AX nuclear fluorescence in UT and Olaparib (6 μM) treated control PEO4 and PEO4 _RPA2_KD cells. H. Cell cycle analysis of UT and Olaparib (6 μM) treated control PEO4 and PEO4_RPA2_KD cells I. Annexin V analysis of UT and Olaparib (6 μM) treated PEO4 and PEO4_RPA2_KD cells. UT = untreated cells; T = cisplatin, talazoparib or olaparib treated cells. The experiment was performed for samples from three independent experiments (n=3) and the error bar represent the standard deviation (SD). ** p<0.01.

**RPA2 depletion promotes platinum sensitivity:** We evaluated basal RPA2 protein levels in platinum resistant (A2780cis and PEO4) and platinum sensitive (A2780, PEO1) ovarian cancer cell lines. As shown in **Supplementary Figures 10A & 10B**, in whole cell lysates, RPA2 was higher in platinum resistant PEO4 and A2780cis cells compared to platinum sensitive PEO1 and A2780 cells respectively. Next, we depleted RPA2 in PEO4 (Fig. 5A). RPA2_KD_PEO4 cells were sensitive to cisplatin compared to platinum resistant controls (Fig. 5B) and was associated with DSB accumulation (Fig. 5C), S-phase cell cycle arrest (Fig. 5D), and increased apoptosis (Fig. 5E). For further validation, we depleted RPA2 in A2780cis cells (**Supplementary Figure. 11A).** RPA2_KD_A2780cis cells were sensitive to cisplatin compared to controls (**Supplementary Figure. 11B)** and associated with DSB accumulation (**Supplementary Figure. 11C)**, S-phase cell cycle arrest (**Supplementary Figure. 11D)**, and apoptosis (**Supplementary Figure. 11E).** We additionally validated using another siRNA construct and observed similar cisplatin cytotoxicity potentiation (**Supplementary Figures 11F& 11G**).

**RPA2 depletion reverses PARP inhibitor resistance:** As shown in Fig. 5F, RPA2_KD_PEO4 cells are sensitive to talazoparib compared to resistant controls which was associated with DSB accumulation (Fig. 5G), S-phase arrest (Fig. 5H) and increased apoptosis (Fig. 5I). Similarly, RPA2_KD_PEO4 cells are also sensitive to olaparib compared to resistant controls (Fig. 5J) which was associated with DSB accumulation (Fig. 5K), S-phase arrest (Fig. 5L) and increased apoptosis (Fig. 5M).

RPA2_KD_A2780cis cells were also sensitive to talazoparib or olaparib compared to controls (**Supplementary Figures 12A & 12E)** and associated with DSB accumulation (**Supplementary Figures 12B & 12F)**, G2/M-phase cell cycle arrest (**Supplementary Figures 12C & 12G)**, and increased apoptosis (**Supplementary Figures 12D & 12H).**

**Fig. 6:**
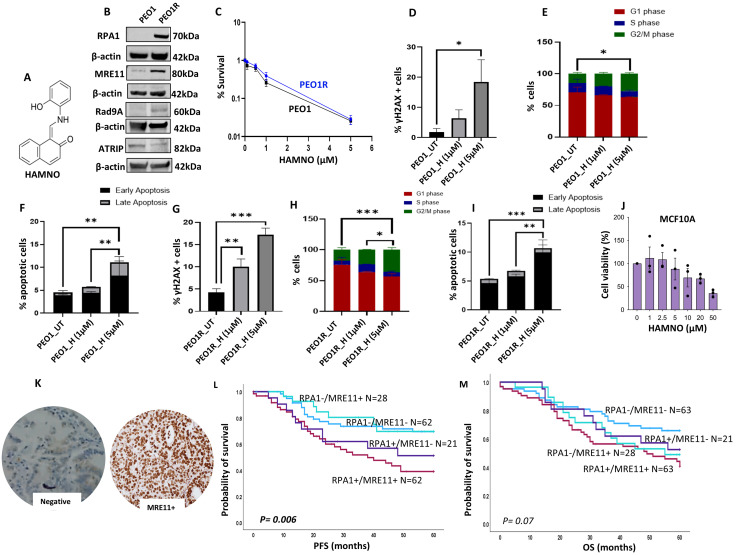
HAMNO sensitivity in PEO1 and PEO1R cells. (A). Chemical structure of HAMNO [((1Z)-1-[(2-hydroxyanilino)methylidene]naphthalen-2-one)]. (B). Western blot of RPA1, MRE11, Rad9, ATRIP in PEO1 and PEO1R cells. (C). Clonogenic assay of HAMNO in PEO1 and PEO1R cells. (D). Quantification of γH2AX nuclear fluorescence in PEO1 cells treated with 1 µM and 5 µM of HAMNO. (E). Cell cycle analysis in PEO1 cells treated with 1 µM and 5 µM of HAMNO. (F). Annexin V analysis of PEO1 cells treated with 1 µM and 5 µM of HAMNO. (G) Quantification of γH2AX nuclear fluorescence in PEO1R cells treated with 1 µM and 5 µM of HAMNO. (H) Cell cycle analysis in PEO1R cells treated with 1 µM and 5 µM of HAMNO. (I). Annexin V analysis of PEO1R cells treated with 1 µM and 5 µM of HAMNO. (J) HAMNO sensitivity in MCF10A cells. (K) Photomicrograph of MRE11 protein expression in human ovarian cancer. (L) Kaplan Meier curves of PFS and RPA1-MRE11 protein co-expression in human ovarian cancer. I. Kaplan Meier curves of OS and RPA1-MRE11 protein co-expression in human ovarian cancer.

**Evaluation of sensitivity to HAMNO monotherapy:** The data presented thus far suggests that RPA blockade could be a potential anti-cancer strategy. The N-terminal domain of RPA1 (70 N) interacts with MRE11, ATRIP, Rad9 and p53 for cell cycle regulation, repair, and apoptosis coordination [[24], [25], [26]]. HAMNO [((1Z)-1-[(2-hydroxyanilino)methylidene]naphthalen-2-one)] is a novel PPI inhibitor that block RPA1- MRE11/ATRIP/p53 interactions [27] (Fig. 6A). Previously we generated PEO1R cells from chronic treatment of PEO1 cells with a MRE11 inhibitor (Mirin) [28]. PEO1R cells are resistant to mirin, cisplatin and PARP inhibitor (olaparib) treatment [28]. Here, western blot analysis revealed that, compared to PEO1, PEO1R cells overexpress RPA1 (Fig. 6B, Supplementary Figure. 13B) and MRE11 (Fig. 6B, Supplementary Figure. 13A). There was no significant difference in Rad9A or ATRIP expression in PEO1 and PEO1R cells (Fig. 6B, Supplementary Figure. 13C & 13D). Therefore, we chose PEO1R pan-resistant cell line to test HAMNO sensitivity and compared to PEO1. As shown in Fig. 6C, PEO1R cells remain sensitive to HAMNO treatment like PEO1 cells in clonogenic assay. In PEO1 cells, increased cytotoxicity was associated with a dose dependent increase in DSB at 1 µM and 5 µM of HAMNO treatment (Fig. 6D), a dose dependent increase in G2/M cells cycle arrest at 1 µM and 5 µM of HAMNO treatment (Fig. 6E) and a dose dependent increase in apoptotic cells at 1 µM and 5 µM of HAMNO treatment (Fig. 6F). Similarly, in PEO1R cells also we observed a dose dependent increase in DSB at 1 µM and 5 µM of HAMNO treatment (Fig. 6G), a dose dependent increase in G2/M cells cycle arrest at 1 µM and 5 µM of HAMNO treatment (Fig. 6H) and a dose dependent increase in apoptotic cells at 1 µM and 5 µM of HAMNO treatment (Fig. 6I). Given the essential functions of RPA in the maintenance of normal cellular homeostasis, we also tested HAMNO in a normal MCF10A breast epithelial cell line. We evaluated toxicity over a range of concentrations (1µM-50 µM). Interestingly, at doses tested in cancer cell lines in the current study (1µM-5 µM), HAMNO showed minimal cytotoxicity to MCF10A (Fig. 6J). The data suggests that a therapeutic window for RPA1-PPI targeting may exist, and cancer cells may be more sensitive to RPA1 targeting compared to normal cells.

**Clinicopathological significance of RPA1/MRE11 co-expression in ovarian cancer:** To understand clinical significance RPA1 co-expression with MRE11, we conducted immunohistochemical evaluation in clinical ovarian cancer cohort. We have recently reported the clinicopathological significance of MRE11 protein expression in human ovarian cancers [29]. A median H-score of >110 was used as the cut-off for high MRE11 nuclear expression (Fig. 6K) in that study [29]. Here, we proceeded to RPA1/MRE11 co-expression analysis. Patients whose tumours had high RPA1 with high MRE11 expression were significantly associated with serous type (p = 0.0001), high grade (p = 0.002), >2 cm residual tumours after surgery (Supplementary Table 2) and poor PFS (p = 0.006) (Fig. 6L). RPA1/MRE11 did not significantly influence OS (P = 0.07) (Fig. 6M).

Taken together, pre-clinical and clinical data suggests that RPA1 PPI inhibition could be a viable strategy in epithelial ovarian cancers.

## Discussion

Replication Protein A (RPA) is a heterotrimeric complex consisting of RPA1, 2, and 3 subunits. RPA is a ssDNA-binding protein that is critically involved in DNA replication, checkpoint regulation and DNA repair. Here we have comprehensively evaluated the clinicopathological significance of RPA1, 2, and 3 in clinical cohorts of ovarian cancer. We show that high expression of RPA1, 2 protein or transcript is linked to aggressive phenotype, platinum resistance and poor survival. Although high *RPA3* transcripts was associated with poor survival, at the protein level RPA3 did not influence survival. Pre clinically, RPA1 or 2 deficient cells are sensitive to cisplatin. Importantly RPA1 or 2 depletion reverses talazoparib or olaparib resistance.

While the functional data strongly suggest a role for RPA in platinum and PARP inhibitor resistance, the clinical cohort does not include patients treated with PARP inhibitors. Together, the data provides evidence that RPA is not only a predictive biomarker of platinum resistance but could also be involved in the development of PARP inhibitor resistance in ovarian cancers. Moreover, a further limitation to our clinical study is that it is retrospective. Moreover, the *RPA* transcript levels were investigated in publicly available data sets and not in the same clinical cohort. We also observed that mRNA expression had a lower prognostic value compared to protein expression. The data suggest that post-translational mechanisms that regulate and/or stabilize RPA protein expression levels may influence prognostic outcome much more than transcript levels. However, platinum resistance is an important surrogate marker of PARPi resistance as these therapies may share overlapping resistance mechanisms [30]. Nevertheless, altered expression of RPA expression has been observed in other solid tumours.

RPA1 and RPA2 overexpression was shown to have poor prognostic significance in patients with colorectal cancer [31]. In grade IV astrocytomas, RPA2 overexpression was associated with poor survival [32]. Elevated RPA1 and RPA2 is associated with advanced stage oesophageal cancers and poor survival [33]. On the other hand, in bladder cancers [34], low RPA1 and low RPA2 was associated with poor survival in patients. Similarly, we have also recently observed that in breast cancers, low RPA1 and low RPA2 was associated with aggressive subtypes and patient outcomes [35]. In our study, high *RPA3* transcripts was associated with poor survival. However, at the protein level, high RPA3 was linked with poor PFS but not OS. The protein-protein interactions (PPI) required for the regulation of DNA replication, repair and recombination is performed through RPA1 (N-terminal domain) and RPA2 (C-terminus) subunits [[11], [12], [13], [14], [15], [16], [17], [18]]. We speculate that this absence of PPI function of RPA3 may limits its predictive/prognostic significance. Taken together, these data suggest a complex clinical role of RPA in cancer pathogenesis.

Standard first-line treatment for newly diagnosed advanced ovarian cancer includes platinum-based chemotherapy (post-operatively or peri-operatively) and maximal debulking surgery if feasible. Despite an initial good response to platinum-based chemotherapy, 70–90 % of patients will develop recurrence and eventually succumb to the disease [36,37]. Although mechanism is complex, upregulation of DNA repair capacity is a common route to platinum resistance. As RPA is known to interact with NER factors (including XPA, XPG, XPF-ERCC1) and FANCJ (that is involved in interstrand cross link repair) [[11], [12], [13], [14], [15], [16], [17], [18]], we speculated that RPA overexpression will increase DNA repair capacity and promote platinum resistance. Depletion of RPA will reduce DNA repair capacity and induce platinum sensitivity. Here we demonstrated that RPA1, 2 overexpression was associated with platinum resistance in clinical cohorts. Pre-clinically RPA1 or 2 depletion restored platinum sensitivity in platinum resistant PEO4 or A2780cis cells. A previous study revealed RPA availability during platinum induced replication stress as an important determinant of cisplatin resistance in ovarian cancer cells [38]. A strong correlation between RPA exhaustion due to aberrant activation of DNA replication origins during replication stress, impaired NER and cisplatin sensitivity was shown in that study. Importantly, RPA overexpression restored NER efficiency and cisplatin resistance [38]. Taken together, the data provides strong evidence that RPA is an important biomarker of platinum resistance in ovarian cancers.

In BRCA germline deficient or HRD or platinum sensitive sporadic ovarian cancers, PARPi, through exploitation of synthetic lethality, have become standard first-line maintenance treatment for ovarian cancers [4]. PARP inhibitors such as talazoparib and olaparib block PARP activity and trap PARP at replication forks resulting in replication stress which eventually lead to DSB accumulation. In cells with HRD due to BRCA2 germ-line mutation, DSBs are unrepaired leading to DSB accumulation which activates programmed cell death [39]. BRCA2 also has roles during processing of stalled replication fork repair and suppression of replication stress. RPA is essential for DNA replication, repair, and recombination [[11], [12], [13], [14], [15], [16], [17], [18]]. Although PARPi maintenance therapy improve progression free survival, 40–70 % of patients will develop resistance to PARPi. Several mechanisms of resistance have been described preclinically [[40], [41], [42], [43]]. In the current study we tested whether PARPi resistance can be reversed in PEO4 cells (BRCA2 revertant) which are derived from PEO1 that harbors *BRCA2* mutation [5193C>G (Y1655X)]. PEO4 is platinum and PARPi resistant HGSOC cell line. Here we demonstrated that RPA depletion can restore PARP inhibitor sensitivity in PEO4 cells. We speculate a model as follows: 1) RPA physically interacts with several factors involved in DSB repair [including 53BP1, BRCA2, ATRIP, DNA-PKcs, MRN complex and others] and DNA recombination [such as RAD51, RAD52, WRN, BLM] [[11], [12], [13], [14], [15], [16], [17], [18]]. 2) RPA depletion in PEO4 will therefore generate a HRD phenotype either by BRCA independent mechanisms or by impairing BRCA-RPA signalling. 3) PARP inhibitors can induce a synthetic lethality phenotype despite a functional BRCA2 in PEO4 cells. As a further validation, we tested platinum resistant A2780cis cells and again demonstrated that RPA1 or 2 depletion induced increased cytotoxicity to PARP inhibitors. An alternative model is also proposed as follows; 1) RPA deficient cells will accumulate single strand DNA breaks (SSB) 2) SSB will activate PARP, a key protein for the coordination of SSB repair 3) PARP blockade by talazoparib/olaparib will not only inhibit PARP biochemical activity but will also trap PARP at replication forks leading onto accumulation of DSB. D) in RPA deficient cells, DSB repair is also impaired resulting in DSB accumulation and cell death. Whilst these potential mechanisms are speculative, detailed functional/mechanistic studies including in vivo model evaluation will be required to confirm the hypothesis.

The multifunctional role of RPA is essential for normal cellular homeostasis. Depletion of RPA such as by siRNA may have far reaching consequences beyond DNA repair. For drug discovery campaigns targeting RPA, therefore, development of inhibitors of protein-protein interactions (PPI) that target only specific pathways is highly desirable to reduce excessive normal tissue toxicity. The N-terminal domain of RPA1 (70 N) interacts with MRE11, ATRIP, Rad9 and p53 for cell cycle regulation, repair, and apoptosis coordination [[24], [25], [26]]. The development of PPI inhibitors that block RPA1-MRE11/ATRIP/Rad9/p53 interactions could be a promising anticancer approach [44]. Isolation of RPA1 small molecule PPI inhibitors have been described recently [reviewed in [26]. HAMNO [((1Z)-1-[(2-hydroxyanilino)methylidene]naphthalen-2-one)] is a prototypical PPI inhibitor that blocks RPA70 - ATR/ATRIP interaction [27]. HAMNO inhibits the checkpoint activation in response to replication stress. HAMNO potentiates etoposide cytotoxicity in vitro and slow tumour growth in vivo [27]. In a recent study, HAMNO was also shown to be selectively toxic in Fanconi anaemia A (FANCA) deficient cells compared to FANCA proficient cells. HAMNO increased cytotoxicity of cisplatin in FANCA-deficient cells compared to FANCA proficient cells [45]. Here we evaluated HAMNO in a resistant cell line model (PEO1R) developed from PEO1 cells after chronic treatment with mirin (a MRE11 inhibitor) [28]. PEO1R overexpressed RPA1 and MRE11, that lead to a highly resistant phenotype. In this model, HAMNO retained sensitivity PEO1R cells like in PEO1 cells. A limitation of pre-clinical study is the therapeutic implications of RPA targeting, particularly with HAMNO, are supported entirely by in-vitro experiments. Although the current data provide strong proof-of-concept, in vivo validation (e.g., xenograft or PDX models using RPA-high platinum-resistant cells) would substantially enhance translational impact and validate our in-vitro observation. Interestingly, TDRL-551, a small molecule blocker of RPA–DNA interaction was also shown to increase platinum cytotoxicity in ovarian cancer in another study [46]. In clinical cohort we showed that ovarian tumours with high levels of RPA1/MRE11 have poor outcomes. In RPA1/MRE11 overexpressing tumours, RPA1- PPI blockade could be a promising approach.

In conclusion, our target validation study suggest that pharmaceutical development of RPA1-PPI inhibitors may be a clinically viable strategy in platinum resistant or PARP inhibitor resistant ovarian cancers.

## Material and methods

### Clinical study

**Patients:** Evaluation of the expression of RPA1, RPA2, and RPA3 in epithelial ovarian cancer (EOC) was performed on tissue microarrays of 331 consecutive cases of EOC treated at Nottingham University Hospitals between 1997 and 2010. Tumour stage was determined according to the International Federation of Obstetricians and Gynaecologists (FIGO) Staging System for Ovarian Cancer. The clinicopathological data included the tumour histology type, International Federation of Obstetricians and Gynecologists (FIGO) stage, grade, and tumour surgical debulking and chemotherapy regimen used. All patients received platinum-based chemotherapy. Platinum resistance was defined as patients who developed progression during first-line platinum chemotherapy or relapse within 6 months after completing platinum treatment. Progression-free survival (PFS) was calculated from the date of the initial surgery to disease progression or from the date of the initial surgery to the last date known to be progression-free for those censored. Overall survival (OS) was calculated from the operation date until the time of death or the last date of follow-up, when any remaining survivors were censored. Patient demographics are summarised in **Supplementary Table 1**.

This study was carried out in accordance with the declaration of The Helsinki and ethical approval which was obtained from the Nottingham Research Ethics Committee (REC Approval Number 06/Q240/153). All patients provided informed signed consent. The Reporting Recommendations for Tumour Marker Prognostic Studies (REMARK) criteria, recommended by McShane et al [47], were followed throughout the study.

**Tissue Microarray (TMA) and Immunohistochemistry (IHC):** Tumour samples were arrayed in tissue microarrays (TMAs) constructed with 2 replicate 0.6 mm cores from the tumours. Immunohistochemical staining was preformed using the Thermo Fisher Scientific Shandon Sequenza chamber system (REF: 72,110,017, Cheshire,UK), in combination with the Novolink Max Polymer Detection System (RE7280-K: 1250 tests, Buffalo Grove, IL, USA), and the Leica Bond Primary Antibody Diluent (AR9352, Buffalo Grove, IL, USA), each used according to the manufacturer’s instructions (Leica Microsystems Buffalo Grove, IL, USA). The TMA slides were deparaffinized with xylene and then rehydrated through five decreasing concentrations of alcohol (100 %, 90 %, 70 %, 50 %, and 30 %) for two minutes each. Pre-treatment antigen retrieval was carried out on the TMA sections using sodium citrate buffer (pH 6.0) and heated at 95 C in a microwave (Whirlpool JT359 Jet Chef 1000 W, UK) for 20 min. A set of slides were incubated with the primary antibodies; RPA1 (Abcam clone ab79398) & RPA2 (Abcam clone ab2175) at a dilution of 1:100 for 60 min at room temperature and RPA3 (Abcam clone ab97436) at a dilution of 1.50 for 60 min at room temperature. Negative controls for IHC included omission of the primary antibody and IgG-matched serum. Positive control included normal lymphoid (Lymph Node/spleen) tissue within the TMA.

**Evaluation of Immune Staining:** The staining was scored using the histochemical score (H-score) system based on both the percentage of immunostaining and the intensity of staining. The staining intensity was assessed as follows: negative staining = 0; weak = 1; moderate = 2; and strong = 3. The percentage of positive tumour cells was estimated in the range of 0–100 %. The H-score was calculated using the formula: (percentage of strongly stained x3 + (percentage of moderately stained x2 + (percentage of weakly stained). TMA cores that were missing because of issues during TMA construction or antigen retrieval or that contained benign tumours, or less than 15 % tumour cells were excluded from scoring. TMA cores that were missing because of issues during TMA construction or antigen retrieval or that contained benign tumours, or less than 15 % tumour cells were excluded from scoring. A median H-score of >140 was used as the cut-off for positive RPA1 nuclear expression, >120 was used as the cut-off for positive RPA2 nuclear expression and a H-score of >20 was used as the cut-off for positive RPA3 nuclear expression.

**Statistical analysis:** Statistical Package for the Social Science (SPSS) software version 22 for Windows (Chicago, IL, USA) was used for statistical analysis. The cut-off points for all RPA complex markers were chosen using the median H-score according to the distribution of the scores for all samples. The univariate associations between the H-scores of the markers and pathological parameters were determined using the Chi-squared test. Survival rates were calculated using Kaplan–Meier survival curves and compared using the log-rank test. *P*-values less than 0.05 were considered statistically significant.

**RPA transcript evaluation***: RPA1, 2 and 3* mRNA expression was evaluated using a publicly available online gene expression dataset for 1287 patients with OC treated with platinum-based chemotherapy from 15 previously published studies that is available at http://kmplot.com/analysis/index.php?p=service&cancer=ovar.

**Bioinformatics:** Analysis of *RPA1, RPA2* and *RPA3* mutations, copy number alterations and mRNA levels on 316 TCGA-OV specimens (TCGA Firehose Legacy) was performed using CBioportal (accessed 07/01/2025 [48]. The TCGA ovarian cancer [49] RNAseq expression data was obtained from GDC (https://portal.gdc.cancer.gov/). The TCGA-OV specimens (n = 379) were ranked from lowest to highest expression for *RPA1, RPA2* and *RPA3*. For the RPA complex calculations, the sum of the ranks of RPA1–3 were taken and then quartiled (low expression levels RPA components Q1 and high expression RPA components Q4). A comparison between Q1 and Q4 was performed to obtain differentially expressed genes between low and high levels of RPA component. The differential analysis utilised DESeq2 [50]. Differential genes of log2 fold of 1 and above, FDR-p value <0.05 were taken forward to pathway analysis using WebGestalt v2019 [51]. Significant pathways showed FDR-p value <0.05. Comparison of differential genes identified for each of the RPA complex was completed using Venny 2.1 [52] (https://bioinfogp.cnb.csic.es/tools/venny/index.html)

### Pre-clinical study

**Compounds:** Cisplatin was kindly provided as a 3.3 mM solution by the pharmacy at Nottingham University City Hospital (Nottingham, UK) and stored at room temperature. Olaparib and Talazoparib were purchased from Selleckchem, UK. The compounds were suspended in 100 % *v/v* dimethyl sulphoxide (DMSO) (276,855–250ML, Sigma Aldrich, UK) at 10 mM and stored at -80°C. HAMNO was purchased from Sigma Aldrich, UK (SML1234).

**Cell lines:** A2780, A2780cis, PEO1 and PEO4 cell lines were purchased from the American Type Culture Collection (ATCC) and cultured as per the ATCC’s recommendations. A2780 (platinum sensitive) cell line was derived from primary human ovarian carcinoma of an untreated patient. A2780cis cisplatin-resistant cell line developed by continuous expose of A2780 cells to doses of cisplatin [53]. PEO1 was Isolated from malignant effusion from peritoneal ascites of a patient with very poorly differentiated serous adenocarcinoma with a *BRCA2* mutation [5193C>G (Y1655X)] PEO4 was derived from the same patient as PEO1 cells after the patient developed resistance to platinum chemotherapy due to restoration of the *BRCA2* mutation [28]. A2780, A2780cis, PEO1 and PEO4 cells were grown in RPMI (1640) media supplemented with 10 % foetal bovine serum (FBS) and 1 % penicillin/streptomycin. The cell lines had been authenticated by the ATCC using the Promega Powerplex® 17 short tandem repeat (STR) system to confirm there was no cross-contamination or misidentification of the cell lines. Mycoplasma testing was routinely performed every month using MycoProbe Mycoplasma Detection Kit (R&D Systems; Abingdon, UK). Cell lines were utilized for experiments for up to 16 passages. MCF10A cells were cultured in DMEM F12 media supplemented with 20 % FBS, 0.4 % BPE, 0.1 % hEGF, 0.1 % insulin, 0.1 % hydrocortisone and 100 ng/mL cholera toxin (Lonzo, CC-4136).

**Western Blot Analysis**: Cells were harvested and lysed in RIPA buffer (R0278, Sigma.UK) with the addition of protease cocktail inhibitor (P8348, Sigma, UK), phosphatase inhibitor cocktail 2 (P5726, Sigma, UK) and phosphatase inhibitor cocktail 3 (P0044, Sigma) and stored at -20°C. Proteins were quantified using BCA Protein Assay kit (23,225, Thermofisher, UK). Samples were run on SDS-bolt gel (4–12 %) bis-tris. Membranes were incubated with primary antibodies as follows: RPA1 (Ab79398, 1:1000, 1 h at room temperature), RPA2 (Ab2175, 1:1000, overnight at 4°C), RPA3 (Ab97436, 1:1000, overnight at 4°C), β-actin (Ab8226, 1:5000, 1 h room temperature), GAPDH (Ab8245,1:1000, 1 h room temperature) and YY1 (Ab109228, 1:2000, 1 h room temperature). Rad9A (1:1000, Cell Signaling 14484S), ATRIP (1:1000, Cell Signaling, 2737S). Membranes then were washed and incubated with Infrared dye-labelled secondary antibodies (LiCor) [IRDye 800CW Donkey Anti-Rabbit IgG (926–32,213) and IRDye 680CW Donkey Anti-Mouse IgG (926–68,072)] at dilution of 1:10,000 for 1 h. Membranes were scanned with a LiCor Odyssey machine (700 and 800 nm) to determine protein levels.

**Nuclear and cytoplasmic extraction:** 2 × 10^6^ cells were seeded into T25 flasks, cultured overnight, and treated with 5 μM of cisplatin for 24 or 48 hours. The cells were collected by trypsinization, centrifuged at 1000 rpm for 5 min at room temperature, and transferred into 1.5 mL Eppendorf tubes. The nuclear and cytoplasmic fractions were separated using the Thermo Scientific NE-PER Nuclear and Cytoplasmic Extraction Kit according to the manufacturer’s instructions (78,833, Thermo Fisher). The extracts were quantified using the BCA protein quantification kit and protein expression levels were checked by western blotting using YY1 as a nuclear marker and GAPDH as a cytoplasmic marker in order to verify the purity of the extractions. Proteins of interest in the nuclear extractions were normalised to YY1. Proteins of interest in cytoplasmic extracts were normalised to GAPDH.

**Transient knockdown (KD) of RPA1 and RPA2:** *RPA1* (two constructs – ID:S12130 and ID:S12132), *RPA2* (two constructs – ID: S12127 and ID: S12128) and negative scrambled control were obtained from Invitrogen (UK). Lipofectamine 3000 reagent (L3000015, Invitrogen, UK) was used according to the manufacturer’s protocol. Briefly cells were seeded at 50–60 % confluency in T25 flasks overnight. Cells were transfected with 20 nM of siRNA oligonuclotide or scrambled siRNA oligonucleotide control (4390,843, Thermofiher) in Opti-MEM media (31,985–062, Gibco). The transfection efficiency was determined at day 3 and day 5 using Western blotting. The knockdown procedures were performed in three independent experiments. Differences between samples were calculated using the Student’s *t*-test.

**Clonogenic assay:** Cells were seeded into six-well plates and allowed to adhere overnight at 37°C in 5 % CO2. The next day, the media was removed, cells were washed with PBS, and 2 mL of fresh media containing the treatments were added. Plates were incubated at 37°C in 5 % CO_2_ for 14 days, then the colonies were fixed, stained, and colonies counted as described above. The survival fraction was calculated as (number of colonies formed after treatment/number of cells seeded) × plating efficiency, using Microsoft Excel 2010. Survival curves were plotted using GraphPad Prism, version 9. Differences between samples were calculated using the Student’s *t*-test. The clonogenic assay were performed in three independent experiments, each in triplicate.

**MTS assay**: MCF10A cells were plated on plastic clear bottomed 96-well plates (2000 cells per well) in complete media. 3 technical repeats were set up for each treatment condition. Plates were incubated at 37°C for 72 hours. Presto blue reagent (Invitrogen A13261) was added to cells in complete media for 1 h at 37°C protected from light. Fluorescence intensity was measured at Ex/Em 520/580–640 nm using a Promega GloMax plate reader. Cell viability data was expressed as percentage viability normalised to DMSO vehicle control.

**Functional studies** 1 × 10^5^ cells per well were seeded in 6- well plates and left overnight at 37°C in a 5 % CO2 atmosphere. After 24 h, 5 µM of Cisplatin or 6 μM olaparib or 800 nM talazoparib were added to cells and incubated for 24 h. Cells then were collected by trypsinization, washed with ice cold PBS and fixed in 70 % ethanol for 1 h at -20°C. After removal of the fixative solution by centrifugation, for DNA double strand break analysis, cells were stained with 2 mg/ml of phospho-Histone (γH2AX) Ser139 (16202A, Millipore, UK). For cell cycle analysis, cells were treated with 20 mg/ml RNase A (12,091,021, Invitrogen) and then 10 mg/ml Propidium Iodide (P4170, Sigma Aldrich) was added to determine the cell cycle distribution. The samples were analysed on a Beckman-Coulter FC500 flow cytometer using a 488 nm laser for excitation and emission data for PI collected using a 620 nm bandpass filter (FL3) and a 525 nm bandpass filter (FL1) for FITC-anti-phospho-Histone H2A.X. For the Apoptosis assay, cells were analysed using Annexin V detection kit (556,547, BD Biosciences). Briefly, cells were trypsinized, washed with PBS and the cellular pellet was re-suspended in Annexin Binding Buffer (1x). Then 2.5 ml of FITC Annexin V and 2.5 ml of Propidium Iodide were added to the cells. After incubation 300 ml of Annexin Binding Buffer (1x) was added to each tube. Samples were analysed on a Beckman-Coulter FC500 flow cytometer. Data were analysed by Weasel software. Graphical representation and statistical analysis were performed in GraphPad Prism 7 (GraphPad, La Jolla, USA).

**Immunofluorescent staining:** 1 × 10^5^ cells per well were plated onto autoclaved coverslips pre-layered in six-well plates, cultured overnight, and treated with cisplatin, olaparib or talazoparib for 24 hours. Briefly, the cells were fixed with 4 % paraformaldehyde (8187,085,000, Sigma Aldrich, UK) for 30 min, permeabilised with 300 μL per well of 0.1 % Triton (85,111, Thermo Fisher Scientific, UK) for 30 min, incubated with 3 % BSA (A7906, Sigma, UK) for 1 h, incubated with 300 μL of diluted primary antibody as follows; 53BP1 [clone:4937S, Cell Signalling, 1:200, overnight at 4°C], γH2AX [clone: JBW301, Sigma, 1:500, overnight at 4°C], Alexa-fluor 488 goat anti-Mouse secondary antibody [clone: A-11,029, 1:200 Thermo Fisher, 1 h at room temperature] and Goat anti-rabbit secondary antibody, TRITC [clone: T-2769, 1:500 Thermo Fisher, 1 h at room temperature]. Then washed with washing buffer (1 % BSA and 0.1 % Tween-20 in PBS) for 3 × 5 min. Next, the cells were incubated with secondary antibodies diluted in 1 % BSA for 1 h in the dark at room temperature, washed with washing buffer (3 × 5 min) and the coverslips were removed using tweezers and placed on clean slides that contained one drop of DAPI (H1200, Vector Laboratories). The coverslip edges were sealed with colourless nail varnish. Imaging was conducted using a Leica SP2 confocal laser scanning microscope. The image analysis was performed using ImageJ software. A minimum of 100 cells per slide were counted for analysis. Data were presented using GraphPad Prism 9 software. The statistical analysis was performed using the Student’s *t*-test.

**Data availability statement:** Data supporting the study can be found in the supplementary information file, and the corresponding author can make any materials available upon request. Aggregate data from the referenced datasets are available from the corresponding author on reasonable request. Primary datasets generated during the study are available in supplementary files 1,2,3 and 4. Referenced datasets analyzed in the study are described in methods and accession codes are as follows; GSE14764, GSE15622, GSE19829, GSE3149, GSE9891, GSE18520, GSE26712, and TCGA.

## Funding statement

This research in Madhusudan laboratory is funded by Naaz-Coker Ovarian Cancer Research Centre, University of Nottingham. Grant number: UoN 17,072,457.

## CRediT authorship contribution statement

**Mashael Algethami:** Writing – review & editing, Writing – original draft, Methodology, Investigation, Formal analysis, Data curation. **Amera Sheha:** Data curation, Formal analysis, Investigation, Methodology, Writing – original draft. **Nehal Singhania:** Writing – review & editing, Writing – original draft, Methodology, Investigation, Formal analysis, Data curation. **Shatha Alqahtani:** Writing – review & editing, Writing – original draft, Methodology, Investigation, Formal analysis, Data curation. **Ahmed Shoqafi:** Writing – review & editing, Writing – original draft, Methodology, Investigation, Formal analysis, Data curation. **Çağla Tosun:** Writing – review & editing, Writing – original draft, Methodology, Investigation, Formal analysis, Data curation. **Jake Spicer:** Writing – review & editing, Writing – original draft, Methodology, Investigation, Formal analysis, Data curation. **Michael S Toss:** Writing – review & editing, Writing – original draft, Methodology, Investigation, Formal analysis, Data curation. **Adel Alblihy:** Writing – review & editing, Writing – original draft, Methodology, Investigation, Formal analysis, Data curation. **Ayat Lashen:** Writing – review & editing, Writing – original draft, Methodology, Investigation, Formal analysis, Data curation. **Jennie N Jeyapalan:** Writing – review & editing, Writing – original draft, Validation, Software, Formal analysis, Data curation, Conceptualization. **Nigel P Mongan:** Writing – review & editing, Writing – original draft, Investigation, Formal analysis, Data curation. **Emad A Rakha:** Writing – review & editing, Writing – original draft, Methodology, Investigation, Formal analysis, Data curation. **Srinivasan Madhusudan:** Writing – review & editing, Writing – original draft, Visualization, Validation, Supervision, Software, Resources, Project administration, Methodology, Investigation, Funding acquisition, Formal analysis, Data curation, Conceptualization.

## Declaration of competing interest

The authors declare that they have no known competing financial interests or personal relationships that could have appeared to influence the work reported in this paper.

## Appendix Supplementary materials

Image, application 1

Image, application 2

Image, application 3

Image, application 4

Image, application 5

Image, application 6

Image, application 7

Image, application 8
