# Supplementary material for: Clinicopathological and functional evaluation of replication protein A in epithelial ovarian cancers: A target validation study
Source: Transl Oncol. 2026 Feb 17;66:102709. doi: 10.1016/j.tranon.2026.102709 (PMC12925576; doi:10.1016/j.tranon.2026.102709)

Full gels

Figure 3A

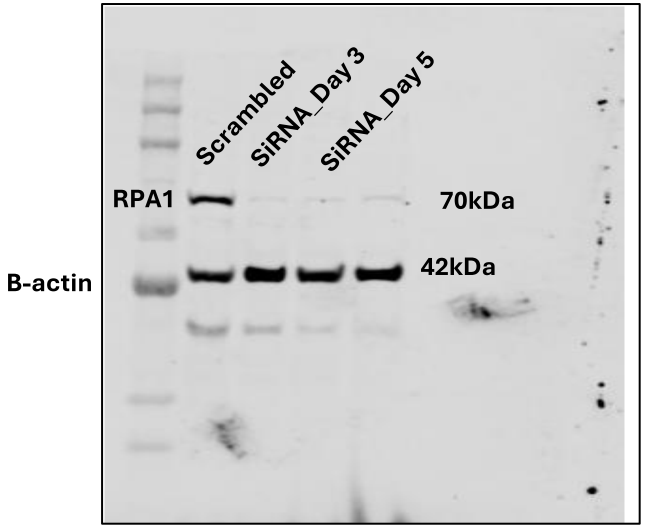

Figure 3H

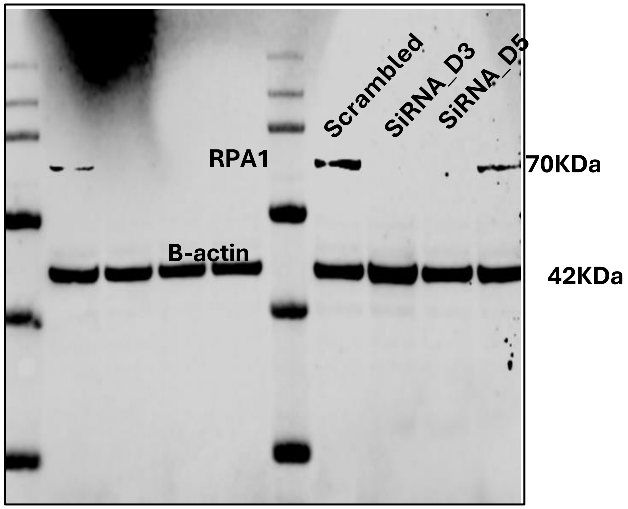

Figure 5A

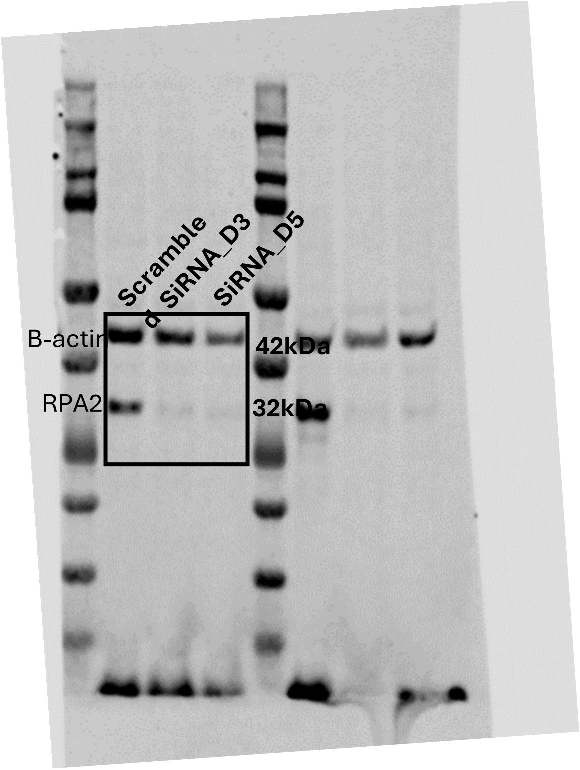

Figure 6B

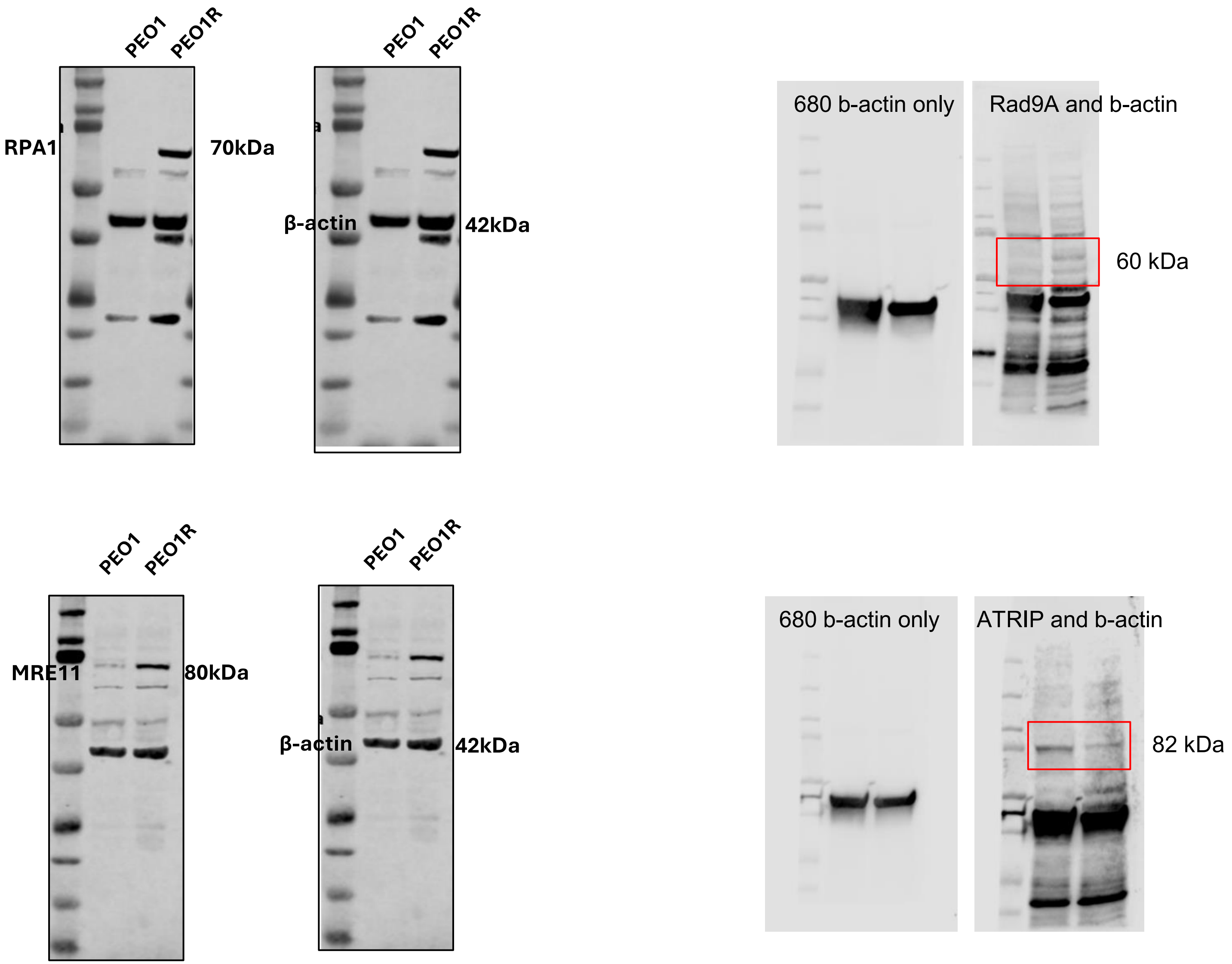
PEO1
PEO1R
RPA1
70kDa
PEO1
PEO1R
β-actin
42kDa
680 b-actin only
Rad9A and b-actin
60 kDa
PEO1
PEO1R
MRE11
80kDa
PEO1
PEO1R
β-actin
42kDa
680 b-actin only
ATRIP and b-actin
82 kDa

Supplementary Figure 2A

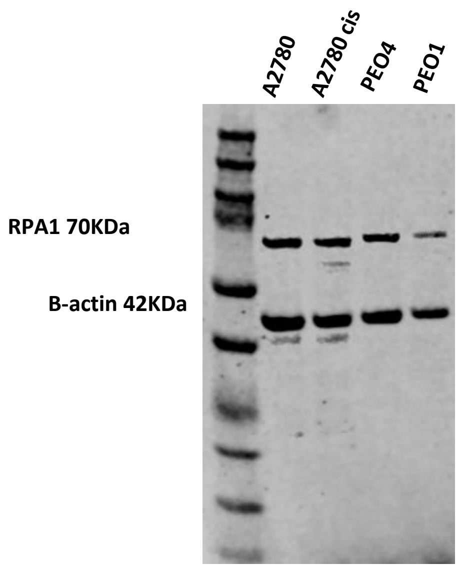

Supplementary Figure 2D

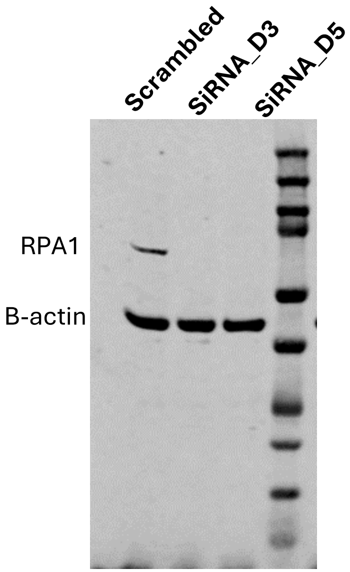

Supplementary Figure 4A

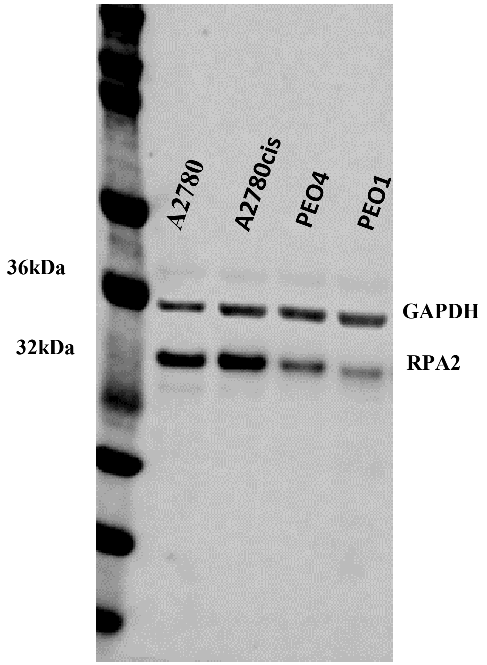

Supplementary Figure 5A

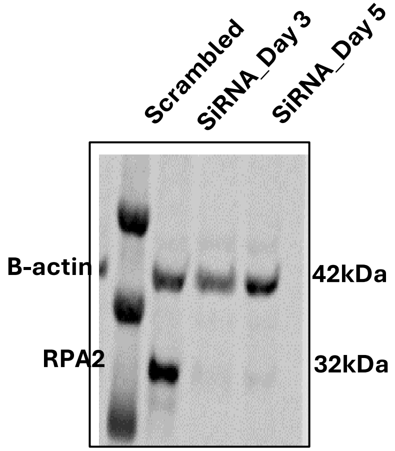

Supplementary Figure 5F

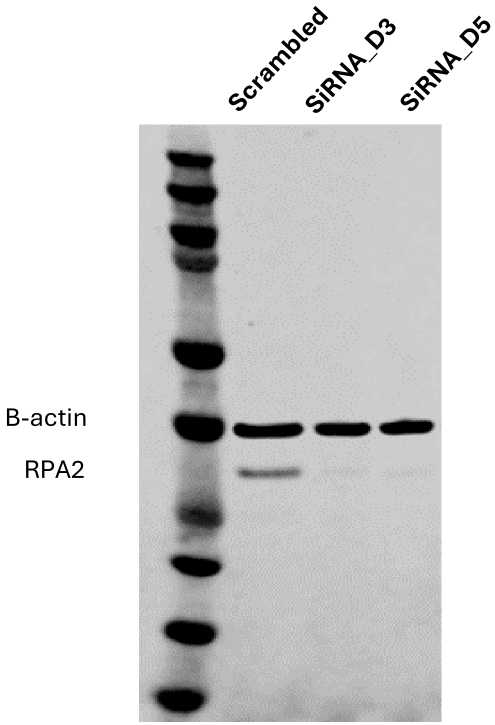

Supplement: Supplementary file 1 [file mmc1.pdf]
