# Supplementary material for: Clinicopathological and functional evaluation of replication protein A in epithelial ovarian cancers: A target validation study
Source: Transl Oncol. 2026 Feb 17;66:102709. doi: 10.1016/j.tranon.2026.102709 (PMC12925576; doi:10.1016/j.tranon.2026.102709)
Supplement: Supplementary file 2 [file mmc2.pdf]

Authorship change request

Important information. Please read before completing this form.
Thisform is torequest any change inauthorship (additions, removals,orreordering) after the submission of a manuscript, including changes in corresponding authors, if any. This form should not be used for changes requested *after* publication or for name changes or corrections.

Prior to completing this form, all authors should carefully review the 'Duties of Authors' section of the Elsevier publishing ethics policy, and in particular, the sections on:

- Authorship of the paper
- The use of generative AI and AI-assisted technologies in scientific writing and in figures, images and artwork

Please also carefully review the journal's guide for authors (this might also be referred to as 'instructions for authors') as some journals may have additional authorship criteria (e.g., the ICMJE guidelines for authorship). The publisher and editor cannot investigate or mediate any authorship disputes. If you are unable to obtain agreement from all authors, including those you intend to remove, we recommend seeking guidance from your institution. We will not consider your change request and will not proceed with the publication of your manuscript until all outstanding authorship disputes are resolved.

If your manuscript is still under consideration, this completed form should be submitted in Editorial Manager for consideration as part of your revision submission (use the 'cover letter' file type).
If your manuscript has already been accepted and is in the proofing or production stages, please return this completed form to the Journal Manager.
If the final version of your manuscript has already been published, a corrigendum will be required. Please see the Article Correction, Retraction and Removal Policy.

Section 1. Submission information

To be completed by the corresponding author.

Submission information	
**Journal title**	Translational Oncology
**Manuscript number and/or article number**	TRANON-D-25-03331
**Manuscript title**	Clinicopathological and functional evaluation of replication protein A in epithelial ovarian cancers: a targe

Change(s) requested (indicate as appropriate)

☑ Add new author(s)	☐ Remove author(s)
☐ Change the corresponding author	☐ Change the order of authors

Section 2. Author(s) added or removed

Completeonetable for eachauthor tobe added orremoved.Please include as much detail as possible in the "Reason for change" section so that we can evaluate if the change is necessary. At a minimum, this should include an explanation for why the change is being requested and why the author was/was not included in the original author list.

If the form is not provided, incomplete, or the reasons provided are insufficiently detailed or do not address the points above, your request will be denied and your submission may be rejected.

Unless instructed by the editor, further changes to the author list without an approved authorship change request will result in the rejection of your submission, or retraction, if the article has already been published.

2.1 Author information

**Given/first name(s)**	Amera Sheha amera.sheha@nottingham.ac.uk Naaz-Coker Ovarian Cancer Research Centre,
**Family/last name**	Biodiscovery Institute, School of Medicine, University o
**Email address**	
**Institution**	
**Change(s) requested** (indicate as appropriate)	☑ Add new author ☐ Remove author ☐ Make the corresponding author

Individual contributions per CRediT Contributor Roles Taxonomy (required for author additions only)

☐ Conceptualization	☑ Data curation	☑ Formal analysis
☐ Funding acquisition	☑ Investigation	☑ Methodology
☐ Project administration	☐ Resources	☐ Software
☐ Supervision	☑ Validation	☐ Visualization
☑ Writing – original draft	☑ Writing – review & editing	

Reason for the change

Dear Editor,
The reviewer comments for the above manuscript required further data analysis.
To address reviewer comments, Dr Amera Sheha had to conduct additional new data curation, methods, investigations, forma analysis and validation. Dr Amera Sheha was incolved in writing the original revision.
We have included Dr Amera Sheha as an additional author to the above manuscript.
Yours Sincerely,
Prof S Madhusudan

2.2 Author information

**Given/first name(s)**	
**Family/last name**	
**Email address**	
**Institution**	
**Change(s) requested** (indicate as appropriate)	☐ Add new author ☐ Remove author ☐ Make the corresponding author

Individual contributions per CRediT Contributor Roles Taxonomy (required for author additions only)

☐ Conceptualization	☐ Data curation	☐ Formal analysis
☐ Funding acquisition	☐ Investigation	☐ Methodology
☐ Project administration	☐ Resources	☐ Software
☐ Supervision	☐ Validation	☐ Visualization
☐ Writing – original draft	☐ Writing – review & editing	

Reason for the change

2.3 Author information

**Given/first name(s)**	
**Family/last name**	
**Email address**	
**Institution**	
**Change(s) requested** (indicate as appropriate)	☐ Add new author ☐ Remove author ☐ Make the corresponding author

Individual contributions per CRediT Contributor Roles Taxonomy (required for author additions only)

☐ Conceptualization	☐ Data curation	☐ Formal analysis
☐ Funding acquisition	☐ Investigation	☐ Methodology
☐ Project administration	☐ Resources	☐ Software
☐ Supervision	☐ Validation	☐ Visualization
☐ Writing – original draft	☐ Writing – review & editing	

Reason for the change

2.4 Author information

Given/first name(s)

Family/last name

Email address

Institution

Change(s) requested (indicate as appropriate) ☐ Add new author ☐ Remove author ☐ Make the corresponding author

Individual contributions per CRediT Contributor Roles Taxonomy (required for author additions only)

☐ Conceptualization	☐ Data curation	☐ Formal analysis
☐ Funding acquisition	☐ Investigation	☐ Methodology
☐ Project administration	☐ Resources	☐ Software
☐ Supervision	☐ Validation	☐ Visualization
☐ Writing – original draft	☐ Writing – review & editing	

Reason for the change

2.5 Author information

**Given/first name(s)**			
**Family/last name**			
**Email address**			
**Institution**			
**Change(s) requested** (indicate as appropriate)	☐ Add new author	☐ Remove author	☐ Make the corresponding author

Individual contributions per CRediT Contributor Roles Taxonomy (required for author additions only)

☐ Conceptualization	☐ Data curation	☐ Formal analysis
☐ Funding acquisition	☐ Investigation	☐ Methodology
☐ Project administration	☐ Resources	☐ Software
☐ Supervision	☐ Validation	☐ Visualization
☐ Writing – original draft	☐ Writing – review & editing	

Reason for the change

*Add additional page(s) as needed for more requested changes.

Section 3. Author order and agreement

Providetheauthor list in theorderthat youwouldlikeit tobepublished.

The form must be signed individually by each author, including any added/removed authors. In cases of consortia group authorship, the corresponding author may sign on behalf of the group.

While handwritten signatures are acceptable, we highly encourage the use of electronic signature software (DocuSign, Adobe Sign, HelloSign, or similar) with valid e-signatures. These signatures should reflect your institutional information and email, as provided in the author list below. Typed signatures or images of signatures will not be accepted.

By signing this form all authors agree:

1) that they have read and acknowledge the publishing ethics policies linked in the "Important Information" section of this form;
2) agree to the addition and/or removal of the authors listed in section 2 and to the revised order of the author list in this section 3, and;
3) that all information provided accurately reflects the authorship of the article.

Agreement of removed author(s)

Full name	Email address	Signature	Date

*Add additional page(s) as needed.

Proposed author list

Order	Fullname	Email address	Signature	Date
01	Mashael Algethami	mashael.a.g@hotmail.com		18/01/2026
02	Amera Sheha	Amera.Sheha@nottingham.ac.uk	Amera Sheha	19-01-2026
03	Nehal Singhania	nehalsinghania@gmail.com		20/1/2026
04	Shatha Alqahtani	shatha.alqahtani@nottingham.ac.uk		18/1/2026
05	Ahmed Shoqafi	ahmed.shoqafi@outlook.sa		19-01-2026
06	Çağla Tosun	Cagla.Tosun1@nottingham.ac.uk		19.01.2026
07	Jake Spicer	Jake.Spicer@nottingham.ac.uk		15-01-26
08	Michael S Toss	michaelsalah85@yahoo.com		18-01-26
09	Adel Alblihy	adel-alblihy@hotmail.com		18/1/2026
10	Ayat Lashen	ayatlashen133@gmail.com	Ayat Lashen	19/01/2026
11	Jennie N Jeyapalan	jennie.jeyapalan@nottingham.ac.uk>		19-01-2026
12	Nigel P Mongan	nigel.mongan@nottingham.ac.uk		15-01-2026
13	Emad A Rakha	Emad.Rakha@nottingham.ac.uk	Emad rakha	19/01/2026
14	Srinivasan Madhusudan	srinivasan.madhusudan@nottingham.ac.uk>		20-01-2026
15				
16				
17				
18				
19				
20				
21				
22				
23				
24				
25				

*Add additional page(s) as needed.
