# Supplementary material for: Clinicopathological and functional evaluation of replication protein A in epithelial ovarian cancers: A target validation study
Source: Transl Oncol. 2026 Feb 17;66:102709. doi: 10.1016/j.tranon.2026.102709 (PMC12925576; doi:10.1016/j.tranon.2026.102709)
Supplement: Supplementary file 3 [file mmc3.docx]

**SUPPLEMENTARY FIGURES**

**
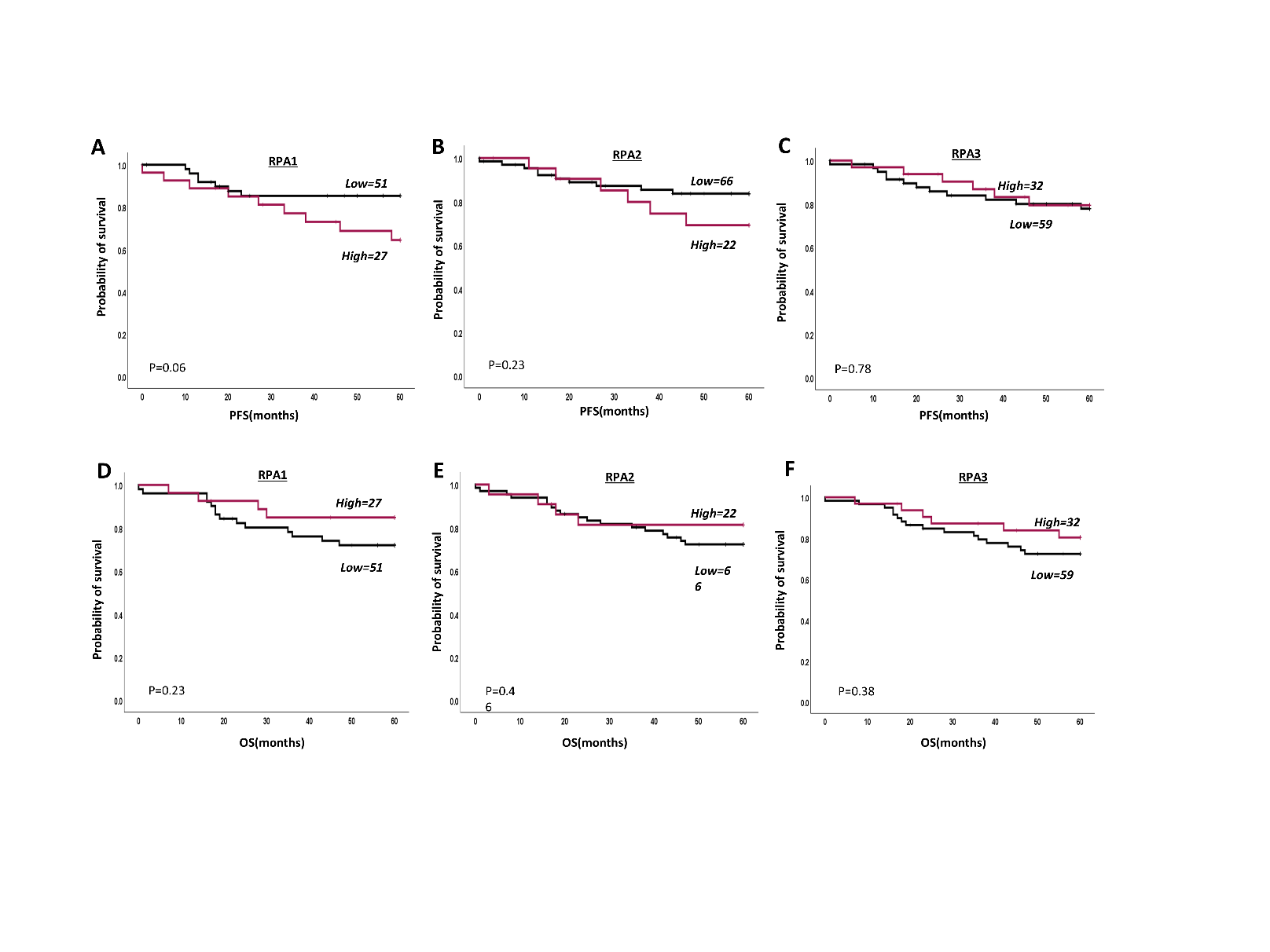
**

**Supplementary Figure 1:** Univariate analysis of RPA1,2 and 3 in stage I ovarian cancer in Nottingham cohort. (A) RPA1 and PFS. (B) RPA2 and PFS. (C) RPA3 and PFS. (D) RPA1 and OS. (E) RPA2 and OS. (F) RPA3 and OS.

**
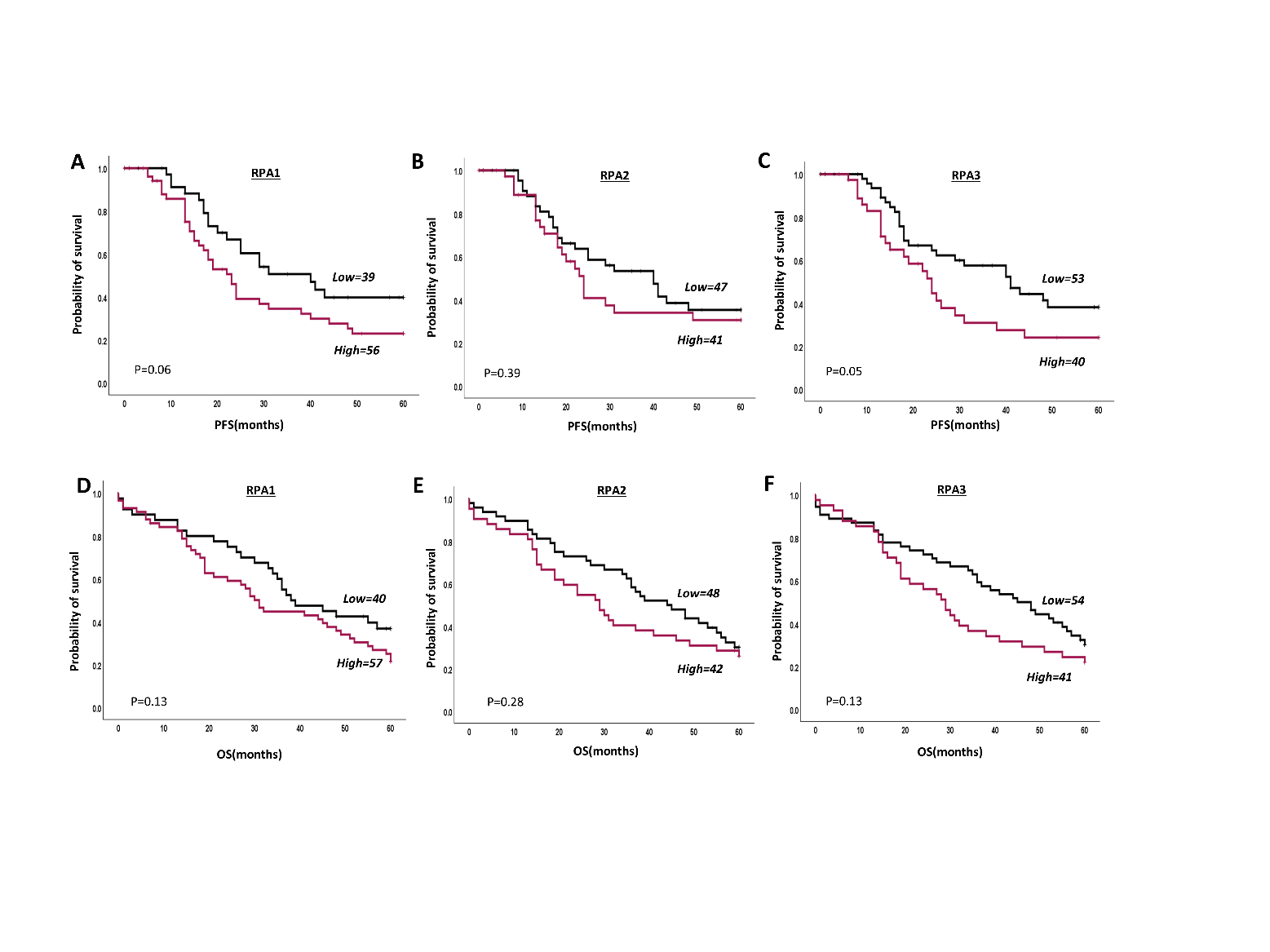
**

**Supplementary Figure 2:** Univariate analysis of RPA1,2 and 3 in stage III ovarian cancer of Nottingham cohort. (A) RPA1 and PFS. (B) RPA2 and PFS. (C) RPA3 and PFS. (D) RPA1 and OS. (E) RPA2 and OS. (F) RPA3 and OS.

**
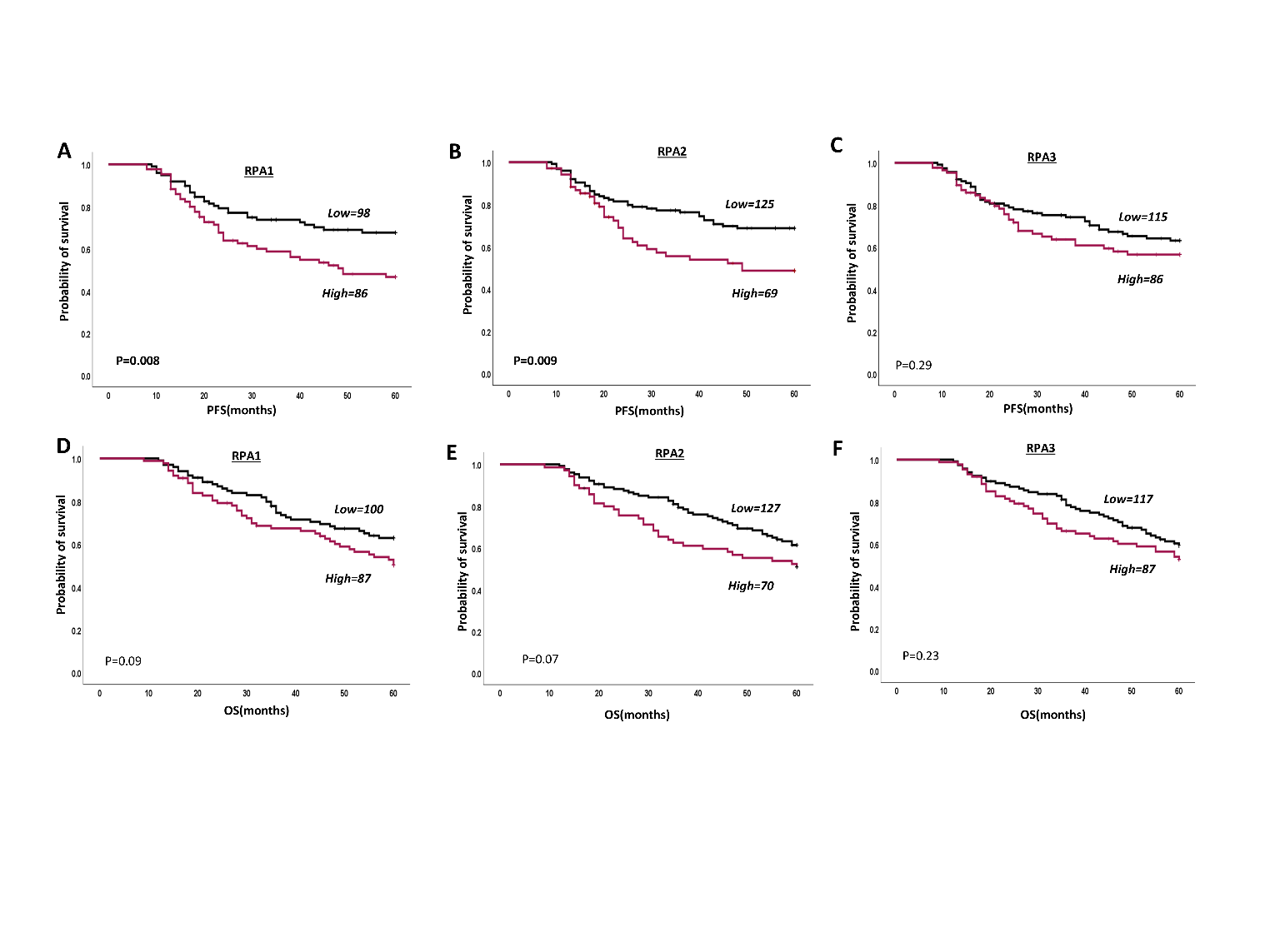
**

**Supplementary Figure 3:** Univariate analysis of RPA1,2 and 3 in platinum sensitive ovarian cancer in Nottingham cohort. (A) RPA1 and PFS. (B) RPA2 and PFS. (C) RPA3 and PFS. (D) RPA1 and OS. (E) RPA2 and OS. (F) RPA3 and OS.

**
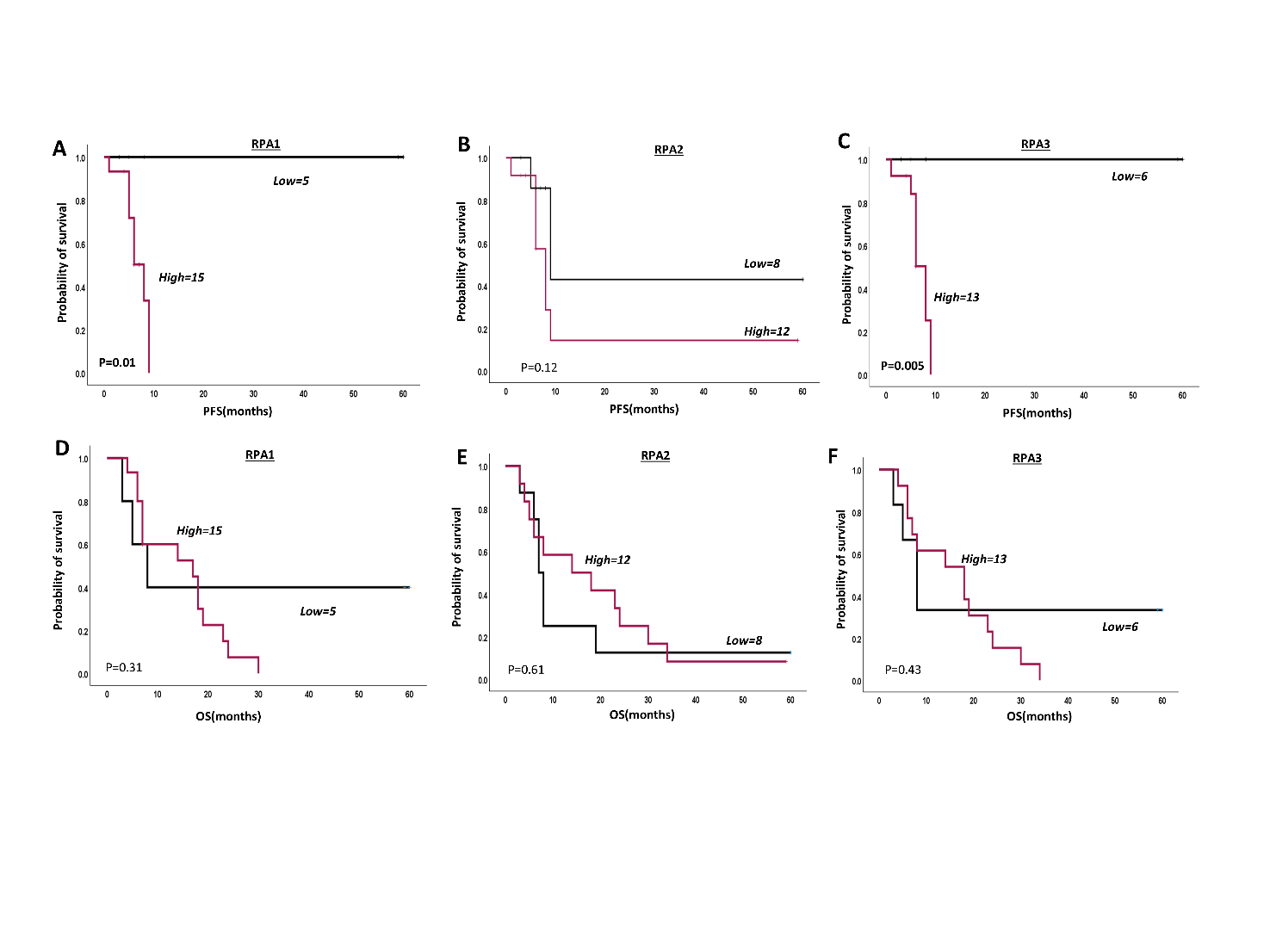
**

**Supplementary Figure 4:** Univariate analysis of RPA1,2 and 3 in platinum resistant ovarian cancer in Nottingham cohort. (A) RPA1 and PFS. (B) RPA2 and PFS. (C) RPA3 and PFS. (D) RPA1 and OS. (E) RPA2 and OS. (F) RPA3 and OS.

**
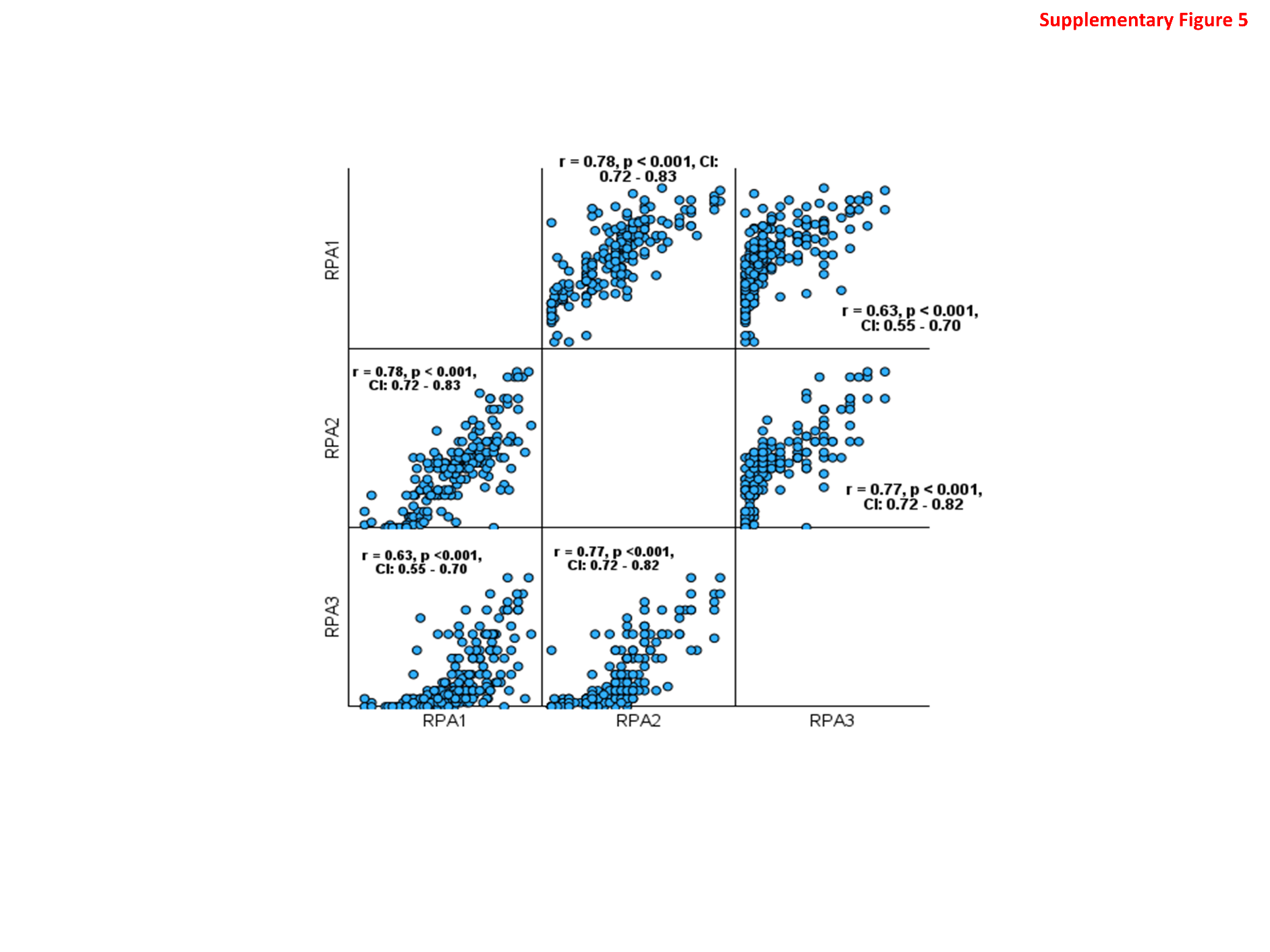
**

**Supplementary Figure 5:** Correlation between RPA protein subunits in Nottingham cohort is shown here. Pearson correlation coefficients (r) with p values and 95% confidence intervals are shown.

**
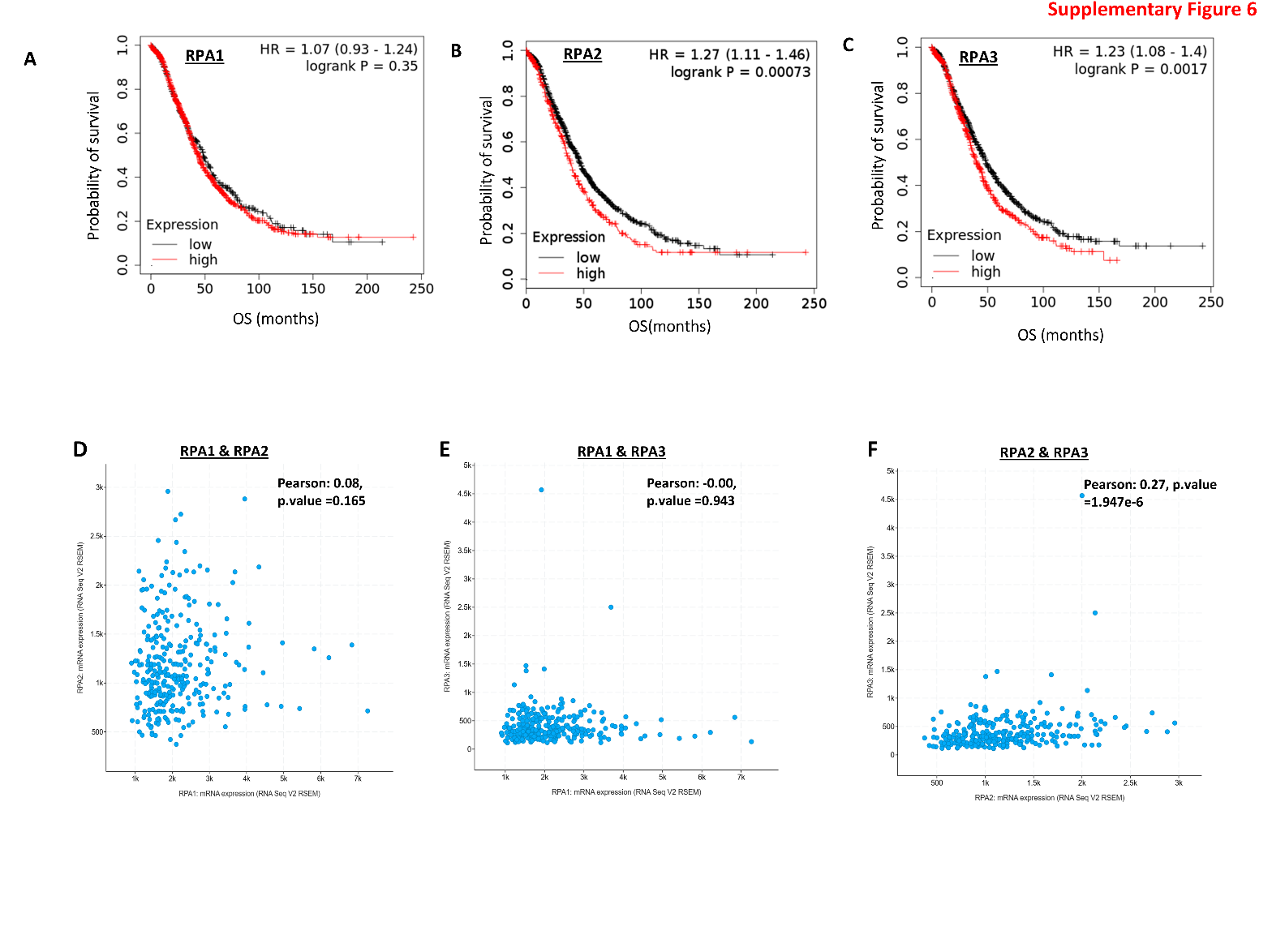
**

**Supplementary Figure 6:** RPA mRNA expression and clinical ovarian cancers. **A.** Kaplan Meier curves of OS and RPA1 mRNA expression. **B.** Kaplan Meier curves of OS and RPA2 mRNA expression. **C.** Kaplan Meier curves of OS and RPA3 mRNA expression. **D-F.** Correlation between RPA subunits at the transcript levels. Pairwise correlations of RPA1 (D), RPA2 (E) and RPA3 (F) mRNA expression across samples from TCGA cohort.

**
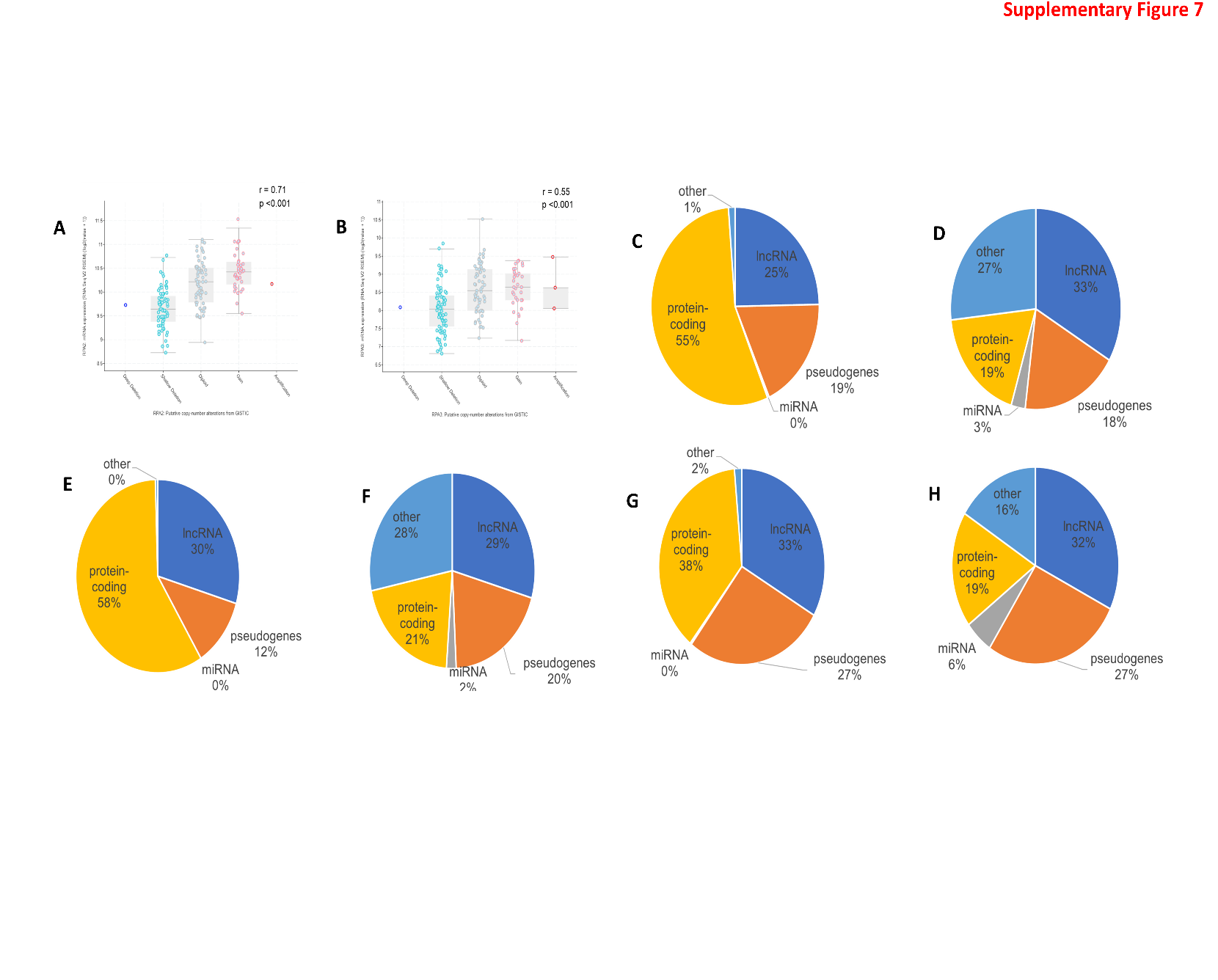
**

**Supplementary Figure 7:** Bioinformatic analysis of genetic alterations and differential gene expression profiling for RPA complex components in TCGA-OV cohort. **A.** GISTIC analysis of *RPA2* genetic alterations compared to mRNA expression levels from RNA-seq data. Pearson correlation coefficient and p-value shown. **B.** GISTIC analysis of *RPA3* genetic alterations compared to mRNA expression levels from RNA-seq data. Pearson correlation coefficient and p-value shown. Graphical representation of the gene-types assigned to the genes expressed higher in Q1 low *RPA1* (n = 1586; **C** & **D**) genes expressed higher in Q4 high *R*PA1 complex (n = 639); **E&F***) RPA2* (Q1 n = 1649 and Q4 n = 316); **G&H**) *RPA3* (Q1 n = 1507; Q4 n = 697). Gene types were lncRNA, miRNA, pseudogenes (including transcribed and processed pseudogenes) and other RNA (tRNA, snoRNA, scaRNA and misc RNA).

**
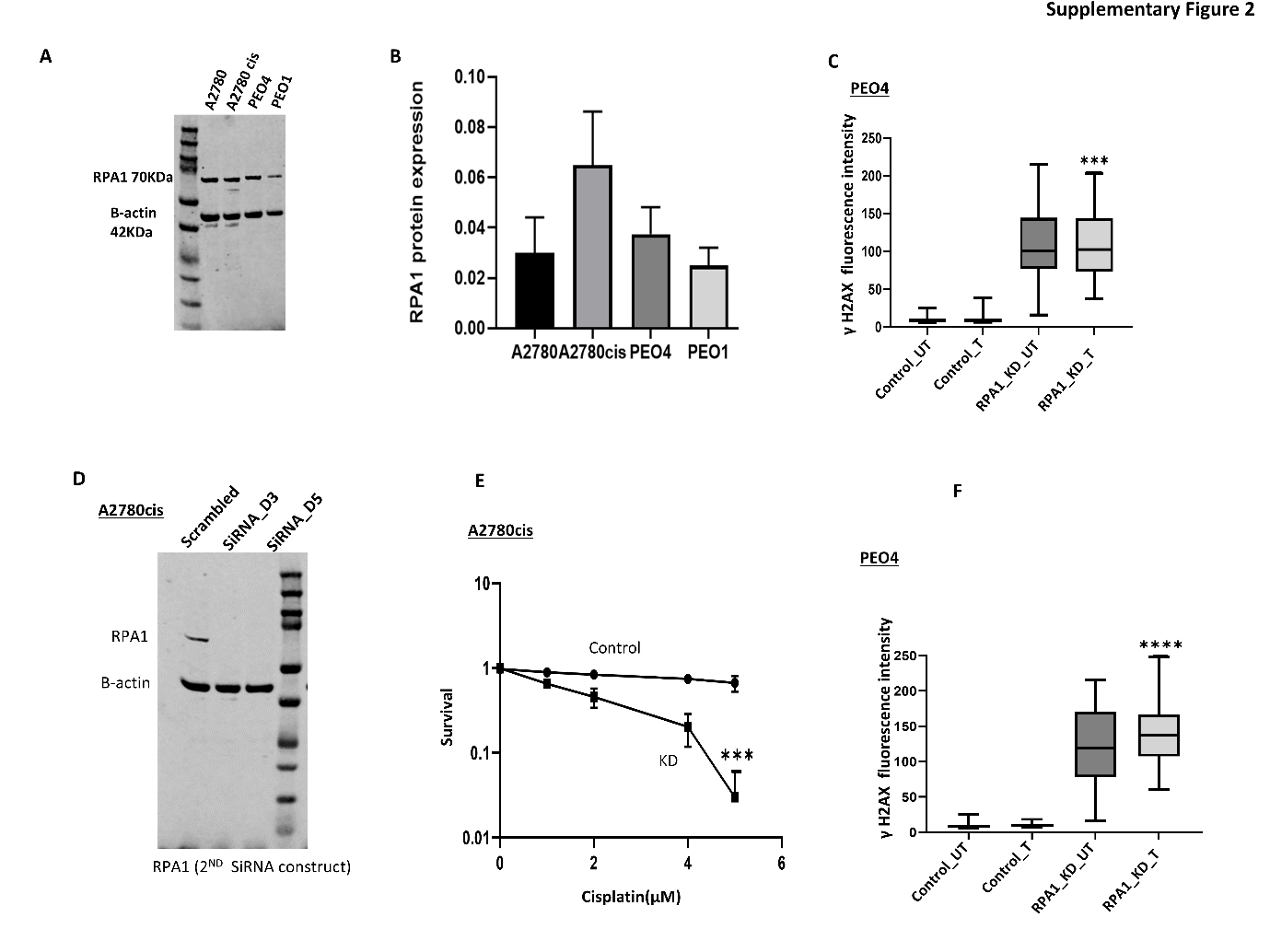
Supplementary Figure 8:** RPA1 protein expression in ovarian cancer cell lines. **A.** Representative western blot of RPA1 protein expression in A2780, A2780cis, PEO4, and PEO1cells. **B.** Quantification of RPA1 levels in A2780, A2780cis, PEO4, PEO1 cells. **C**. Quantification of γH2AX nuclear fluorescence in UT and cisplatin (5 µM) treated control PEO4 and PEO4 _RPA1_KD cells. **D.** Representative western blot of RPA1 knock down (KD) using second siRNA construct in A2780cis cells. **E.** Clonogenic assay of cisplatin sensitivity in control A2780cis and RPA1_KD_ A2780cis cells. **F.** Quantification of γH2AX nuclear fluorescence in UT and talazoparib (800 nM) treated control PEO4 and PEO4 _RPA1_KD cells

**
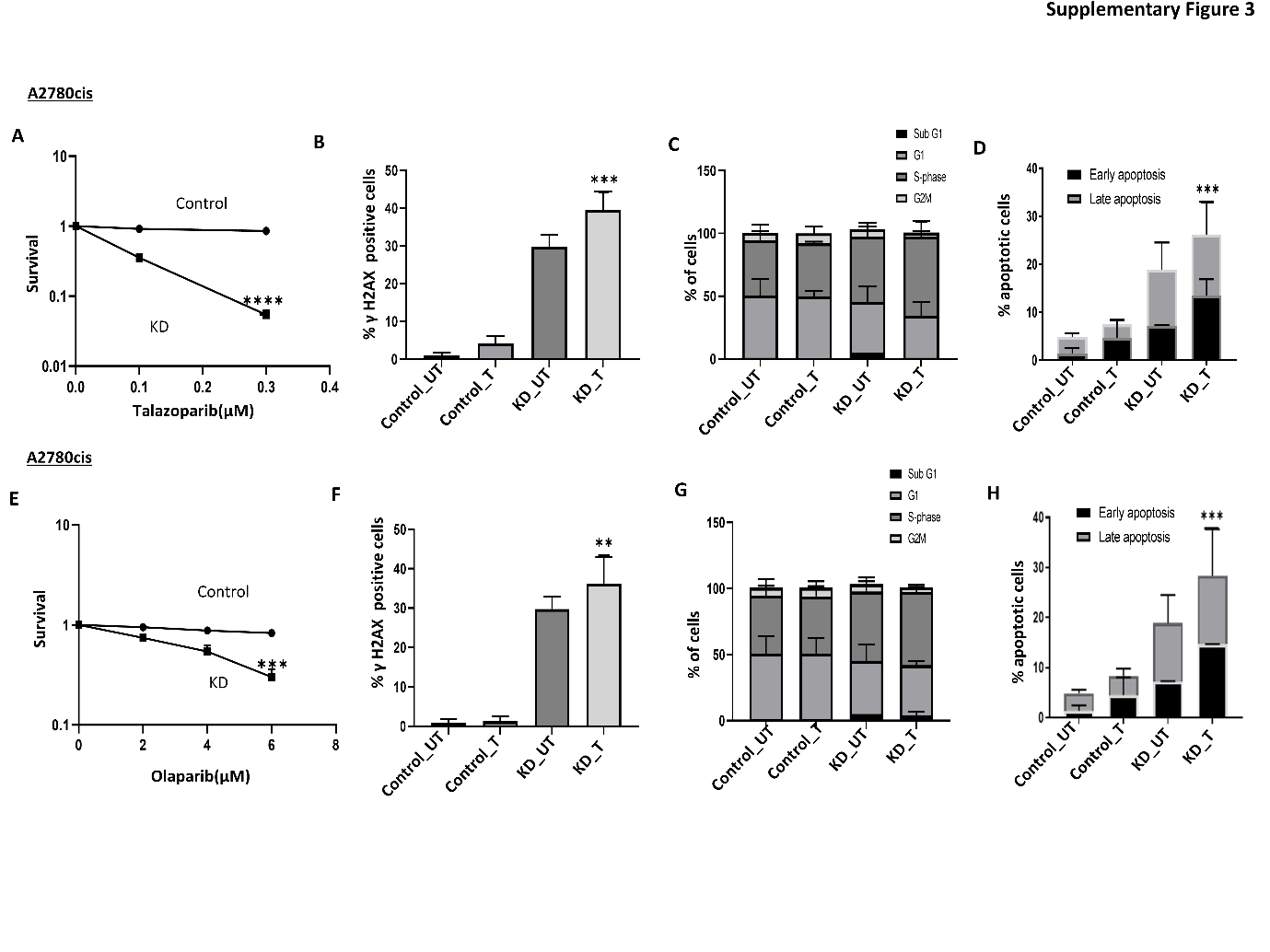
Supplementary Figure 9:** RPA1 depletion and PARP inhibitor sensitivity in A2780cis cells. **A.** Clonogenic assay of talazoparib sensitivity in control A2780cis and RPA1_KD_A2780cis cells. **B.** Quantification of γH2AX nuclear fluorescence in UT and talazoparib (800 nM) treated control A2780cis and A2780cis _RPA1_KD cells. **C.** Cell cycle analysis of UT and talazoparib (800 nM) treated control A2780cis and A2780cis _RPA1_KD cells **D.** Annexin V analysis of UT and talazoparib (800 nM) treated A2780cis and A2780cis _RPA1_KD cells. **E.** Clonogenic assay of olaparib sensitivity in control A2780cis and RPA1_KD_ A2780cis cells. **F.** Quantification of γH2AX nuclear fluorescence in UT and Olaparib (6μM) treated control A2780cis and A2780cis _RPA1_KD cells. **G.** Cell cycle analysis of UT and Olaparib (6μM) treated control A2780cis and A2780cis _RPA1_KD treated cells. **H.** Annexin V analysis of UT and Olaparib (6μM) treated control A2780cis and A2780cis _RPA1_KD treated cells. UT = untreated cells; T = talazoparib or olaparib treated cells. The experiment was performed for samples from three independent experiments (n=3) and the error bar represent the standard deviation (SD). ** p<0.01.

**
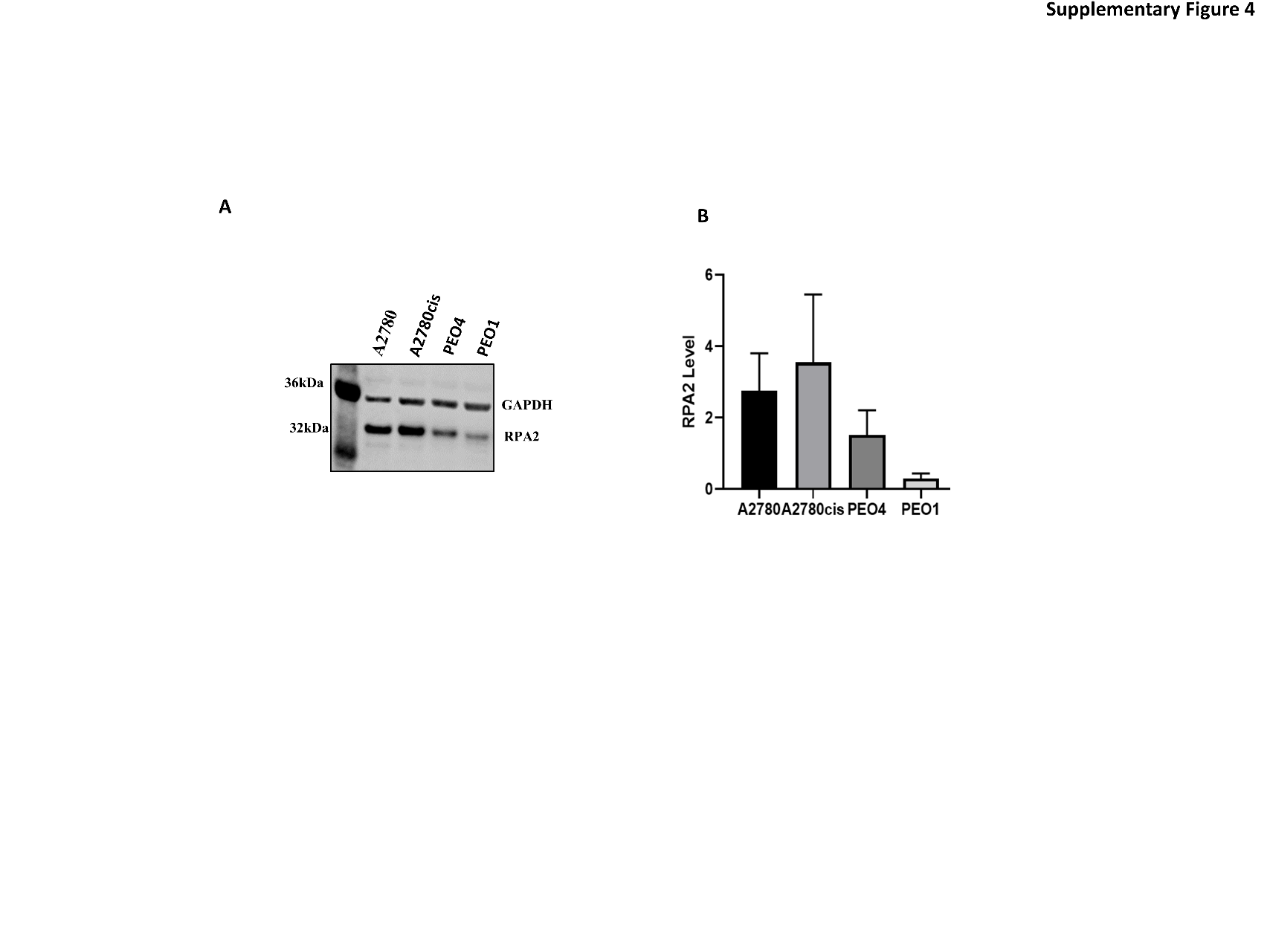
Supplementary Figure 10:** RPA2 protein expression in ovarian cancer cell lines. **A.** Representative western blot of RPA2 protein expression in A2780, A2780cis, PEO4, and PEO1cells. **B.** Quantification of RPA2 levels in A2780, A2780cis, PEO4, PEO1 cells.

**
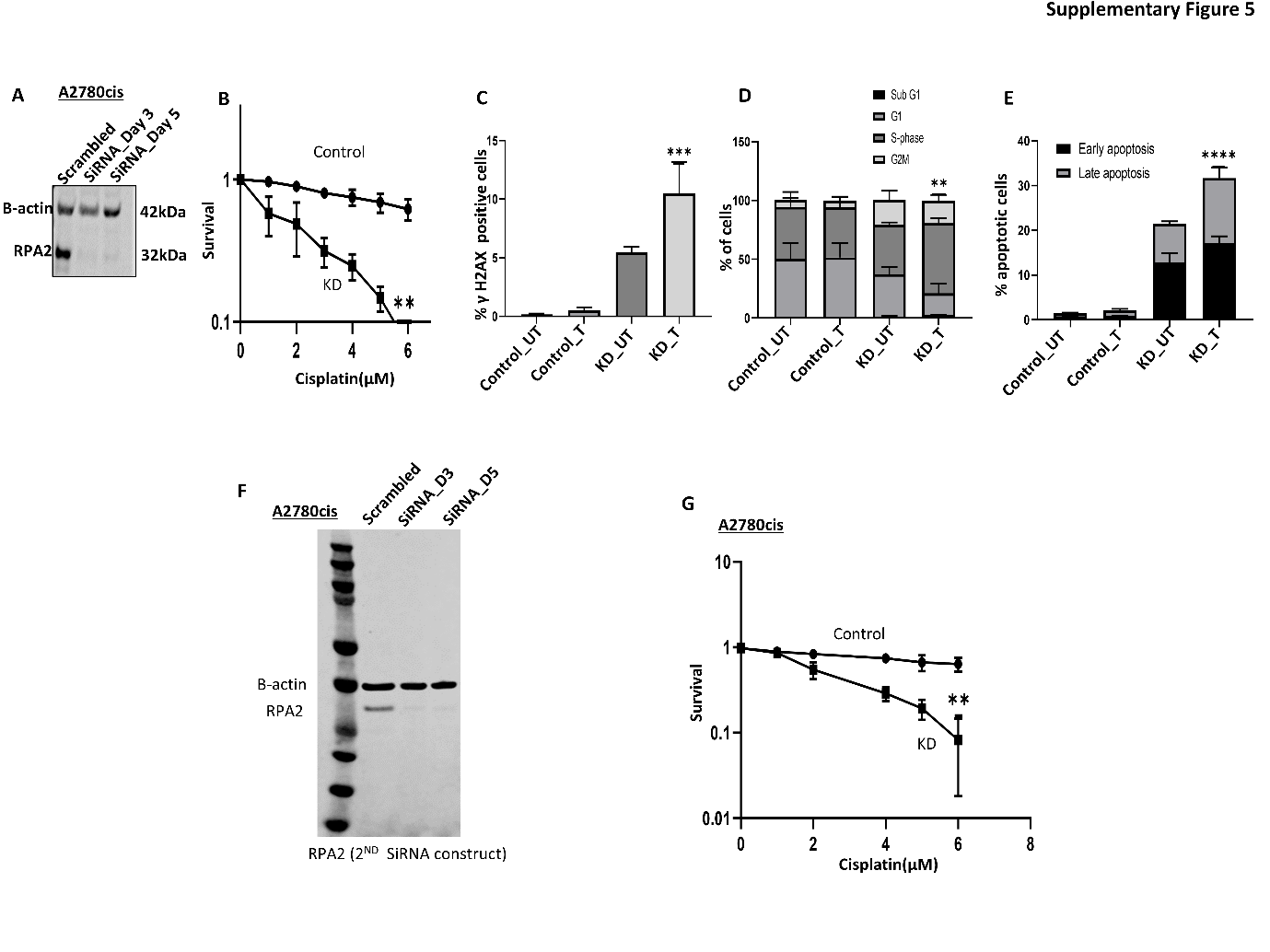
Supplementary Figure 11:** RPA2 depletion and cisplatin or PARP inhibitor sensitivity. **A.** Representative western blot of RPA2 knock down (KD) using siRNA in A2780cis cells. **B.** Clonogenic assay of cisplatin sensitivity in control A2780cis and RPA2_KD_ A2780cis cells. **C.** Quantification of γH2AX nuclear fluorescence in UT and cisplatin (5μM) treated control A2780cis and A2780cis _RPA2_KD cells. **D.** Cell cycle analysis of UT and cisplatin (5μM) treated control A2780cis and A2780cis _RPA2_KD cells **E.** Annexin V analysis of UT and cisplatin (5μM) treated A2780cis and A2780cis _RPA2_KD cells. **F.** Representative western blot of RPA2 knock down (KD) using second siRNA construct in A2780cis cells. **G.** Clonogenic assay of cisplatin sensitivity in control A2780cis and RPA2_KD_ A2780cis cells.

**
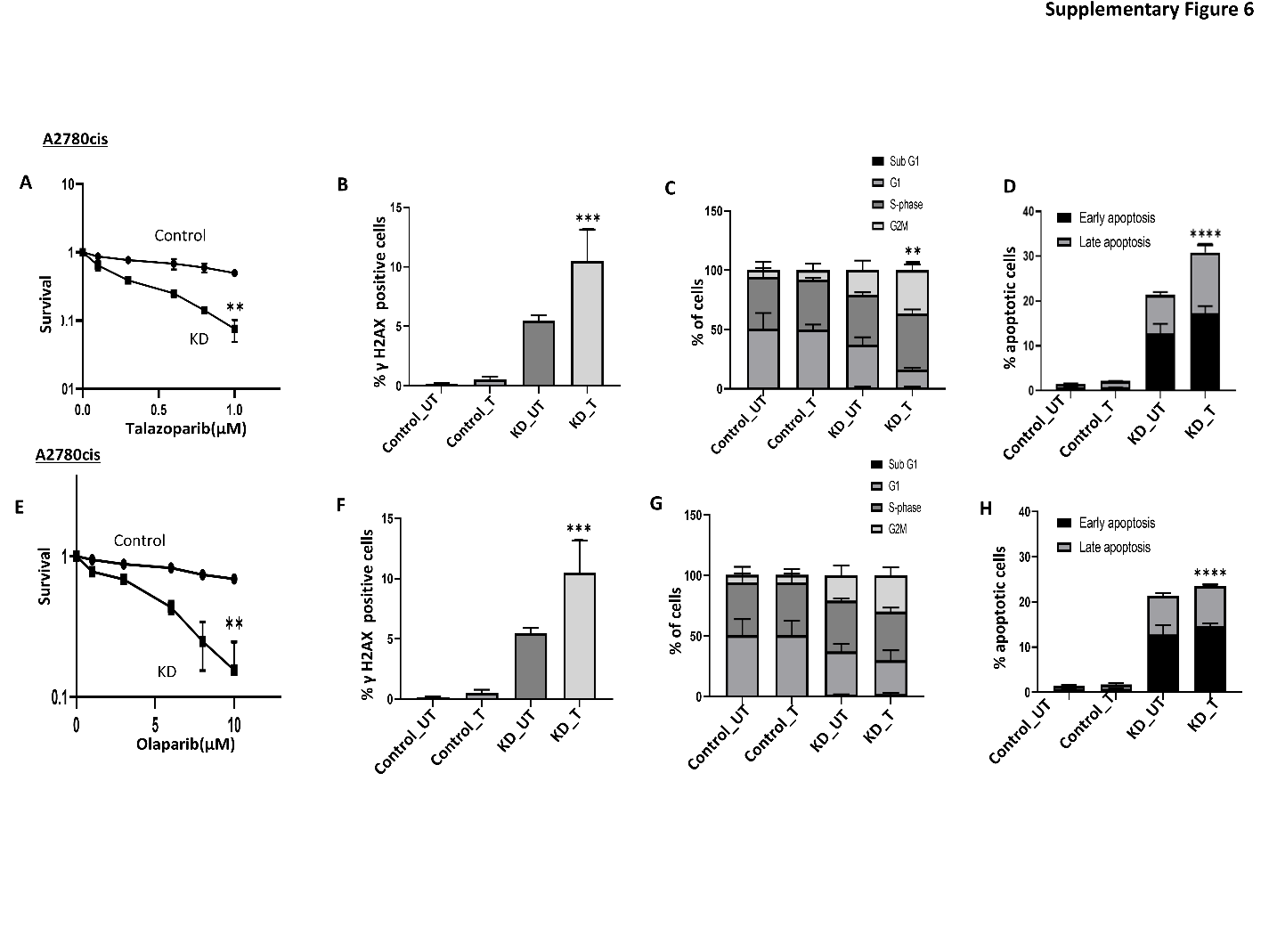
Supplementary Figure 12:** RPA2 depletion and PARP inhibitor sensitivity in A2780cis cells. **A.** Clonogenic assay of talazoparib sensitivity in control A2780cis and RPA2_KD_ A2780cis cells. **B.** Quantification of γH2AX nuclear fluorescence in UT and talazoparib (800 nM) treated control A2780cis and A2780cis _RPA2_KD cells. **C.** Cell cycle analysis of UT and talazoparib (800 nM) treated control A2780cis and A2780cis _RPA2_KD cells **D.** Annexin V analysis of UT and talazoparib (800 nM) treated A2780cis and A2780cis _RPA2_KD cells. **E.** Clonogenic assay of Olaparib sensitivity in control A2780cis and RPA2_KD_ A2780cis cells. **G.** Quantification of γH2AX nuclear fluorescence in UT and Olaparib (6μM) treated control A2780cis and A2780cis _RPA2_KD cells. **H.** Cell cycle analysis of UT and Olaparib (6μM) treated control A2780cis and A2780cis _RPA2_KD cells **I.** Annexin V analysis of UT and Olaparib (6μM) treated A2780cis and A2780cis _RPA2_KD cells. UT = untreated cells; T = cisplatin, talazoparib or olaparib treated cells. The experiment was performed for samples from three independent experiments (n=3) and the error bar represent the standard deviation (SD). ** p<0.01.

**
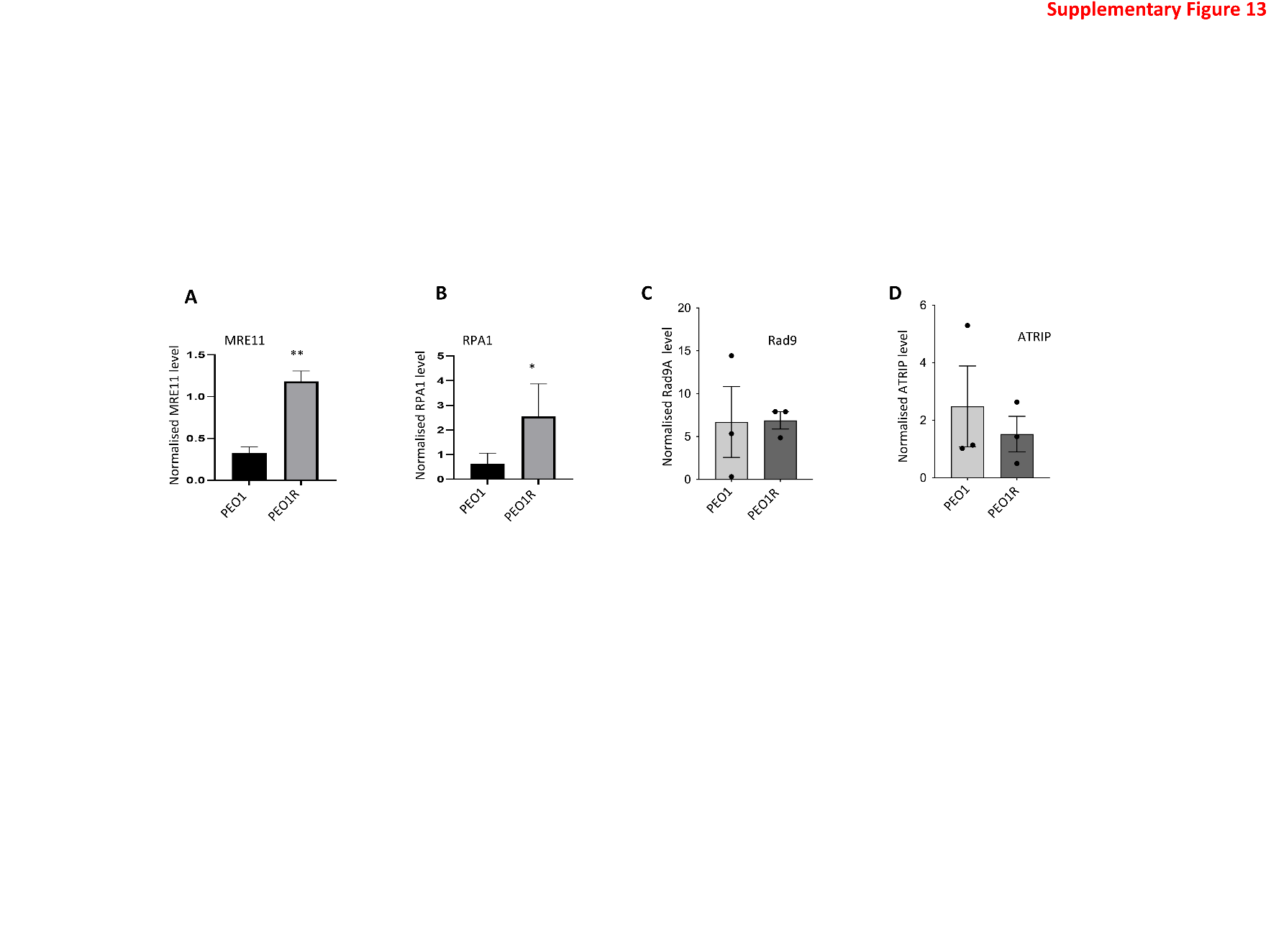
**

**Supplementary Figure 13:** Protein quantification by western blotting in PEO1 and PEO1R cells of **(A)** MRE11, **(B)** RPA1, **(C)** Rad9 and **(D)** ATRIP
