# Supplementary material for: Clinicopathological and functional evaluation of replication protein A in epithelial ovarian cancers: A target validation study
Source: Transl Oncol. 2026 Feb 17;66:102709. doi: 10.1016/j.tranon.2026.102709 (PMC12925576; doi:10.1016/j.tranon.2026.102709)
Supplement: Supplementary file 8 [file mmc8.docx]

**Supplementary Table 1:** Patient demographics and pathological features in ovarian cancer cohort.

| **Characteristics** | | **Number** | **Percentages** |
| --- | --- | --- | --- |
| ***Pathology*** | | | |
|  | Serous cystadenocarcinoma | 178 | 53.9% |
|  | Endometrioid | 44 | 13.2% |
|  | Clear cell carcinoma | 24 | 7.2% |
|  | Mucinous cystadenocarcinoma | 46 | 13.8% |
|  | Others | 15 | 4.5% |
|  | Mixed | 17 | 5.1% |
|  | missing | 7 | 2.3% |
| ***Grade*** | | | |
|  | 1 | 46 | 13.8% |
|  | 2 | 60 | 18% |
|  | 3 | 171 | 51.5% |
|  | missing | 54 | 16.7% |
| ***Residual tumors*** | | | |
|  | None/Microscopic | 205 | 61.7% |
|  | <1cm | 34 | 10.2% |
|  | >1-2 cm | 15 | 4.5% |
|  | >2cm | 40 | 12% |
|  | Missing | 37 | 11.6% |
| ***FIGO Stage*** | | | |
|  | I | 123 | 37% |
|  | II | 49 | 14.7% |
|  | III | 128 | 38.5% |
|  | IV | 11 | 3.3% |
|  | Missing | 20 | 6.5% |
| ***Chemotherapy*** | | | |
|  | Carboplatin monotherapy | 100 | 30% |
|  | Carboplatin + Paclitaxel | 110 | 33% |
|  | Other regimens * | 26 | 8% |
|  | No chemo | 23 | 7 % |
|  | unknown | 72 | 22% |
| ***Platinum sensitivity*** | | | |
|  | Sensitive ** | 250 | 75.6% |
|  | Resistant | 26 | 7.8% |
|  | Unknown | 55 | 16.6% |
| ***Relapse status*** | | | |
|  | Progression-free *** | 169 | 50.9% |
|  | Progressed/relapsed | 118 | 35.6% |
|  | Unknown | 44 | 13.3% |

*Other Regimens* included: 3= CAP (Cyclophosphamide, Adriamycin and Cisplatin) 4 = ICON5 Trial 5 = SOCTROC Trial 6 = Carboplatin and Endoxan. 7 = Chlorambucil. For the unknown category, the majority received platinum-based chemo but because the exact regimen was not mentioned clearly in the database, they were considered as unknown. However, for these cases the platinum sensitivity status were clearly mentioned / recorded.

**Platinum resistance was defined as patients who had progression during first-line platinum chemotherapy or relapse within 6 months after completion of platinum treatment.

*** Progression-free survival was calculated from the date of the initial surgery to disease progression or from the date of the initial surgery to the last date known to be progression-free at the end of follow-up. All the patients’ dates of disease recurrence were recorded in the main sheet where we used to perform the analysis.

**Supplementary Table 2.** RPA1-MRE11 co-expression and ovarian cancers

|  | ***Low RPA1/low MRE11***  ***N (%)*** | ***High RPA1/low MRE11***  ***N (%)*** | ***High RPA1/high MRE11***  ***N (%)*** | ***Low RPA1/high MRE11***  ***N (%)*** | ***P value*** |
| --- | --- | --- | --- | --- | --- |
| **Histological Type** |  |  |  |  |  |
| Serous | 24 (36%) | 17 (77%) | 59 (80%) | 20 (67%) | **0.000005** |
| Mucinous | 18 (27%) | 2 (10%) | 1 (2%) | 1 (3%) |  |
| Endometriod | 12 (18%) | 1 (4%) | 7 (10%) | 6 (20%) |  |
| Clear Cell | 8 (12%) | 0 (0%) | 1 (2%) | 1 (3%) |  |
| Other | 2 (3%) | 2 (9%) | 3 (4%) | 0 (0%) |  |
| Mixed | 4(6%) | 0 (0%) | 3 (4%) | 2 (7%) |  |
| **Tumour Grade** |  |  |  |  |  |
| Low | 14 (24%) | 0 (0%) | 6 (9%) | 2 (8%) | **0.008** |
| Med | 14 (24%) | 2 (11%) | 13 (19%) | 9 (35%) |  |
| High | 30 (52%) | 17 (89%) | 50 (72%) | 15 (57%) |  |
| **Tumour Stage** |  |  |  |  |  |
| 1 | 35 (53%) | 7 (33%) | 19 (25%) | 19 (33%) | **0.031** |
| 2 | 8 (12%) | 3 (14%) | 15 (21%) | 5 (17%) |  |
| 3 | 22 (33%) | 11 (53%) | 38 (52%) | 12 (40%) |  |
| 4 | 1 (2%) | 0 (0%) | 2 (2%) | 3 (10%) |  |
| **Platinum Sensitivity** |  |  |  |  | 0.199 |
| Sensitive | 59 (97%) | 20 (95%) | 52 (87%) | 26 (93%) |  |
| Resistant | 2(3%) | 1 (5%) | 8 (13%) | 2 (7%) |  |
